# Aggregation operators on group-based generalized q-rung orthopair fuzzy N-soft sets and applications in solar panel evaluation

**DOI:** 10.1016/j.heliyon.2024.e27323

**Published:** 2024-03-06

**Authors:** Muhammad Saeed Raja, Khizar Hayat, Adeeba Munshi, Tahir Mahmood, Rawish Sheraz, Iqra Matloob

**Affiliations:** a Department of Mathematics, University of Kotli, Azad Jammu and Kashmir, 11100, Pakistan; b Department of Mathematics & Statistics, International Islamic University, Islamabad, Pakistan

**Keywords:** N-soft sets, q-ROFNSS, GGq-ROFNSS, Decision making, Aggregation operators

## Abstract

Every problem in decision-making has a solution when the information that is available is properly and precisely modeled. This study focuses on non-binary data from N-soft sets and q-rung orthopair fuzzy values, referred to as group-based generalized q-rung orthopair fuzzy N-soft sets (GGq-ROFNSSs). The GGq-ROFNSSs model provides information simultaneously on numerous competing criteria, alternatives, sub-alternatives, and data summarization. We introduce properties of GGq-ROFNSSs such as distinct inclusion features of GGq-ROFNSSs, weak complements of the GGq-ROFNSS, top weak complements the GGq-ROFNSS, bottom weak complements the GGq-ROFNSS. We provide the notion of GGq-ROFNSWA and GGq-ROFNSWG operators as well as their idempotency, monotonicity, and boundedness features. The notion of GGq-ROFNSSs requires a sound methodology of multiple criteria decision making (MCDM) since GGq-ROFNSS combines numerous elements of complex decision-making. We provide a MCDM methodology for the GGq-ROFNSWA and GGq-ROFNSWG operators and depict it in a flowchart. The selection of solar panels for a city is a difficult procedure because it depends on several components such as environment, where the area is located, what kinds of needs are being met, etc. We find a solution to the problem of selecting a suitable solar panel for a city with their underlying characteristics. Finally, we provide a comparison of the suggested method with other techniques to demonstrate its advantages.

## 1 Introduction

In order to obtain better conclusion and decision, the decision-making process must always be put forward. Making decisions involves a sophisticated brain process that considers numerous elements in an effort to arrive at a desirable result. This process may be rational or illogical; on the other hand, it may make implicit or explicit assumptions that are influenced by a wide range of factors, including cultural, physiologic, social, biological and industrial effects. The complexity of a decision-making process can vary depending on all of these elements, as well as endorsement and risk. Nowadays, complex decision-making problems can be resolved using algebra, a variety of statistical and mathematical equations, some theories of economic, and computer technologies that calculate and estimate the answers to decision-making questions automatically.

Fuzzy sets (FSs) can be used to deal with the uncertainty and ambiguity that are present in many systems and processes used nowadays for decision-making. In order to deal with multiple uncertainties in challenging MCDM scenarios, Zadeh developed the concept of FSs [1]. Although membership in FSs enhanced the precision of the value of a statement's object, more was still required when hesitation was taken into consideration. To further refine the concept of a FS, Atanassov [2] created the intuitionistic fuzzy set (IFS) theory [3]. In 2013 Yager point out restrictive circumstances of IFSs by introducing the idea of Pythagorean fuzzy sets (PFSs) [4], [5]. However, there was still a remaining space where IFSs and PFSs are failed to cope credible and non-credible information. Thus there were a requirement of more general structure, Yager in 2016 cover this deficiency by initiating q-rung orthopair fuzzy sets (q-ROFSs) [6]. Ali [7], Garg [8] and Peng [9] discussed several properties of q-ROFSs. Different kinds of q-ROFSs, including exponential-logarithm q-ROFSs [10], complex q-ROFSs [11], and linguistic q-ROFSs [12], have been developed recently. In MCDM approaches, fuzzy aggregation operators play a significant role in combining vast amounts of data [3], [13], and the same is true for q-ROFSs in order to get superior results [8], [9], [11].

Molodtsov [14] developed a theoretical framework known as soft sets (SSs) that relied on approximations of attributes rather than fuzzy grades to evaluate uncertainties. In contrast to conventional set theory, SS theory is a mapping in which the approximation of the attribute in the domain is a subset of a universal set in range. Maji et al. [15] and Ali et al. [16] both defined certain fundamental SS operations and basic properties. Although the SS model is a smooth tool for parameter estimations, fuzziness can still be seen in SS‘s approximations. Maji et al. [17] identified fuzzy SSs (FSSs), which addressed both parametrization and ambiguity in circumstances of MCDM. In recent decay hybrid models of FSSs, intuitionistic fuzzy SSs (IFSSs) [18], q-ROF soft sets (q-ROFSSs) [19], [20], and parameterized possibility fuzzy soft sets [21] have been popularized. Different types of operators and properties on q-ROFSSs have been introduced by several researchers; Hussian et al. [22], [23] expressed q-ROFSS aggregation operators on Dombi t-norm and t-conorm, Zulqarnain et al. [24] expressed q-ROFSS Einstein operators, Ali et al. [25] expressed bipolarity of q-ROFSS, and Salsabeela et al. [26] introduced a new MCDM on q-ROFSSs.

In practice, the rating and assessment of objects is typically represented by the quantity of points and stars. For example the quality of any object is denoted as follows, poor quality; one star, improved quality; two stars, good quality; three stars, well quality; four stars, greatest quality. Fatimah et al. [27] expanded the idea of soft set theory to address this challenge, introduced the N-soft set model, and discussed the value of ordered grades in real-world problems with non-binary evaluation environments. Riaz et al. [28] introduced some fundamental operations on N-soft set and the idea of N-soft topology. In many cases non-binary evaluation environments are also blinded with grades of membership. On this motivation, Akram et al. [29] introduced fuzzy N-soft set and their operation laws. The N-soft sets have been applied in parameter reduction and rough sets [30], [31]. The decision-making process can be improved by combining fuzzy soft sets and fuzzy N-soft sets. Fuzzy N-soft sets allow us to consider several levels of membership or non-membership, which can give decision-makers more robust and thorough information. In fuzzy and soft set-based decision issues, fuzzy N-soft sets may provide decision makers more information as compared to sole binary systems.

One of the main challenges and disadvantages of fuzzy N-soft sets is that they only examine the assignment of membership degrees to the parameterized characterization of the elements by several experts, ignoring the potential of independent assessments on non-membership degrees. It should be noted that only the parameterized object's membership degrees are taken into account by the fuzzy N-soft set; its non-membership degrees are not taken into consideration. By combining IFSs with N-soft sets, Akram and Ali [32] introduced the intuitionistic fuzzy N-soft set (IFNSS) theory to address this flaw. Zhang et al. [33] and Akram et al. [34] developed the idea of Pythagorean fuzzy N-soft set (PFNSS) by joining PFS and N-soft set and applied it to MCDM problems. The q-rung orthopair fuzzy N-soft sets (q-ROFSSs) and q-ROFSS aggregation operators have been proposed by Zhang et al. [35]. They illustrated the effectiveness and practicality of q-ROFSSs in decision-making by investigating a practical application. Spherical fuzzy N-soft expert sets and dual hesitant N-soft sets have recently been introduced and used in MCDM [36], [37].

Generalized intuitionistic fuzzy soft sets (GIFSSs), a hybrid model of IFSS developed by Agarwal et al. [38], is a significant method for expressing judgment about IFSS while accounting for the viewpoint of a senior expert (moderator). In order to address the shortcomings of the GIFSS in Agarwal et al. [38]), Feng et al. [39] confirmed the concept and presented operations on the GIFSSs. When group-based GIFSS (GGIFSS) was developed by Garg and Arora [40], the idea introduced by Agarwal et al. [38] was expanded upon by taking into account the evaluation of more than one moderator. A different form of the notion of GGIFSS was introduced by Hayat et al. [41], and various revisions to the idea of GIFSSs were made by altering aggregation operator phenomena.

A new type of aggregation operator on GGIFSSs was recently proposed by Hayat et al. [42], who defined new aggregation instruments on GGIFSSs and provided more reliable findings than existing operators. This framework represents the data in terms of expert team judgments in the form of membership and non-membership values, as well as it summaries data on particular possibilities. With several decision-makers or experts involved, the group-based component enables the aggregation of individual preferences or opinions. The technique described in Hayat et al. [42] takes into account an example of choosing a hand sanitizer utilizing the MCDM approach on aggregations operators. A unique VIKOR approach for generalized PFSSs and its application to young children in COVID quarantine were introduced by Kirisci et al. in 2022 [43]. Group-based generalized q-ROFSSs (GGq-ROFSSs) and their uses in MCDM were later outlined by Hayat et al. [44]. The idea of GGq-ROFSSs presented in Hayat et al. [44] offers a lot of potential for solving MCDM issues [45]. The MCDM using GGq-ROFSSs offers a methodical approach to deal with uncertainties in cases when alternatives and criteria depend on the final determinations of senior experts and the weights of each component in GGq-ROFSSs are significant. Recently, interval valued GGq-ROFSSs and new aggregation operators have been introduced [46]. In Hayat et al. [46], the goal of GGq-ROFSSs is established by taking into consideration a sport MCDM example, which demonstrates the importance of each component of GGq-ROFSSs. On these, motivation there is a research gap in considering N-soft sets and additional parameters in the same model. The GGq-ROFSSs is a complete model to label all aspects of MCDM; therefore, considering q-RONFSSs and additional parameters is a more suitable approach to handle MCDM problems.

In this work, the concept q-ROFNSS presented in the article [35] is explored in term of Hayat et al., [42], [44], [46]. This work's primary motivation is to take binary systems of membership and non-membership grades into consideration, along with various levels of ratting. Studying q-ROFSs with N-SSs is crucial for MCDM problems when non-binary systems are required. First, we introduce N-parameterized q-rung orthopair fuzzy set (N-Pq-ROFS) and then using idea of N-Pq- ROFS and q-ROFNSS we define group generalized q-ROFNSS (GGq-ROFNSS).

This concept not only considers non-binary systems but also those additional scenarios which are significant in MCDM problems, (i) teamwork evaluations as q-ROFNSSs, (ii) data summarization for a specific as N-Pq-ROFSs. According to the logical MCDM point of view, this notion may have a wide range of applications in areas such as industrial and biological decision-making, artificial intelligence, and recommendation systems, among others. We define GGq-ROFNSS complement, GGq-ROFNSS bottom complement and GGq-ROFNSS top complement. By considering tabular representation of GGq-ROFNSS, we investigated basic ideas such as q-ROFN number of GGq-ROFNSS. Then we introduce GGq-ROFNSWA and GGq-ROFNSWG operators and their underlying properties. The choice of solar panels for a city is a challenging process since it depends on the environment, the location of the area, the sector of needs, etc. [47], [48]. Consequently, we consider the MCDM method on GGq-ROFNSSs, and then we illustrate an MCDM problem for solar panels selection in a city. The methods of Hayat et al. [46] and Hussian et al. [22] are compared with proposed method of GGq-ROFNSWA and GGq-ROFNSWG operators.

## 2 Preliminaries

In this section, we recall basic ideas including soft sets (SSs), N-soft sets (NSSs), q-rung orthopair fuzzy sets (q-ROFSs), q-rung orthopair fuzzy N-soft sets (q-ROFNSSs), etc. In this article, a collection of alternatives will be referred to a set U.

### 2.1 q-rung orthopair fuzzy sets

Yager investigated Pythagorean fuzzy sets (PFSs) and q-ROFSs which are vital generalizations of IFSs. The q-ROFSs are composed of a wider set of scenarios with credible information (membership) and non-credible information (non-membership).

Definition 2.1 [6], [7] A q-ROFS in a universe U is defined as

(1) $$F=\{(u,\phi_{F}(u),\varphi_{F}(u))|u\in U\},$$

where the functions $\phi_{F}:U\to [0,1]$ and $\varphi_{F}:U\to [0,1]$ respectively assign the degree of membership grade and the degree of non-membership grade of the element u∈U. Further, it is required that $0\leq {\left(\phi_{F}(u)\right)}^{q}+{\left(\varphi_{F}(u)\right)}^{q}\leq 1$ ∀u∈U, where q≥1. The hesitancy degree of q-ROFS is indicated as $\pi_{F}(u)=\sqrt[{q}]{1-({\left(\phi_{F}(u)\right)}^{q}+{\left(\varphi_{F}(u)\right)}^{q})}$.

The set of all q-ROFSs over U is denoted by $q-ROFS^{U}$. For any b∈U, q-ROF number (q-ROFN) is expressed by ϰ=〈ϕ(b),φ(b)〉. For simplicity, we denote q-ROFN by ϰ=〈ϕ,φ〉. In order to transfer q-ROFN into real value number, the score function is utilized and given as in following definition.

Definition 2.2 [7], [49] Let $\varkappa_{1}=\langle \phi ,\varphi \rangle$ be a q-ROFN. Then the score function and accuracy function of *ϰ* can be expressed as $S(\varkappa )=\phi^{q}-\varphi^{q}$ and $L(\varkappa )=\phi^{q}+\varphi^{q}$, respectively.

Now consider two q-ROFNs $\varkappa_{1}=\langle \phi ,\varphi \rangle$ and $\varkappa_{2}=\langle \phi^{\prime },\varphi^{\prime }\rangle$. Then

i If $S(\varkappa_{1})>S(\varkappa_{2})$, then $\varkappa_{1}>\varkappa_{2}$.
ii If $S(\varkappa_{1})=S(\varkappa_{2})$, then;
  • if $L(\varkappa_{1})>L(\varkappa_{2})$, then $\varkappa_{1}>\varkappa_{2}$,
  • if $L(\varkappa_{1})=L(\varkappa_{2})$, then $\varkappa_{1}=\varkappa_{2}$.

Some crucial operations on q-ROFNs are given as in the following notion.

Definition 2.3 [49] Let $\varkappa_{1}=\langle \phi ,\varphi \rangle$ and $\varkappa_{2}=\langle \phi^{\prime },\varphi^{\prime }\rangle$ be two q-ROFNs, with *δ* being a positive real number. Consequently, the fundamental operations are given as;

• $\varkappa_{1}⊕\varkappa_{2}=\langle \sqrt[{q}]{{\left(\phi \right)}^{q}+{\left(\phi^{\prime }\right)}^{q}-{\left(\phi \right)}^{q}{\left(\phi^{\prime }\right)}^{q}},\varphi \varphi^{\prime }\rangle$,
• $\varkappa_{1}⊗\varkappa_{2}=\langle \phi \phi^{\prime },\sqrt[{q}]{{\left(\varphi \right)}^{q}+{\left(\varphi^{\prime }\right)}^{q}-{\left(\varphi \right)}^{q}{\left(\varphi^{\prime }\right)}^{q}}\rangle$,
• $\delta \varkappa =\langle \sqrt[{q}]{1-{\left(1-{\left(\phi \right)}^{q}\right)}^{\delta }},\varphi^{\delta }\rangle$,
• $\varkappa^{\delta }=\langle \phi^{\delta },\sqrt[{q}]{1-{\left(1-{\left(\varphi \right)}^{q}\right)}^{\delta }}\rangle$,
• $\varkappa_{1}\cup \varkappa_{2}=\langle max\{\phi ,\phi^{\prime }\},min\{\varphi ,\varphi^{\prime }\}\rangle$,
• $\varkappa_{1}\cap \varkappa_{2}=\langle min\{\phi ,\phi^{\prime }\},max\{\varphi ,\varphi^{\prime }\}\rangle$,
• $\varkappa^{c}=\langle \varphi ,\phi \rangle$.

### 2.2 q-rung orthopair fuzzy N-soft sets

In this section we recall the idea of NSSs, q-ROFNSSs and their fundamental operations.

Definition 2.4 [14], [16] Let U be a collection of objects and C is a set of attributes, B⊆C. The soft set is the mapping from B to power set P(U), that is, S:B→P(U).

Definition 2.5 [27] Let U be a collection of objects and C is a set of attributes, B⊆C. Let P={0,1,2,⋯,N−1} be a set of ordered grades, here N={2,3,⋯}. A triple (S,B,N) is a N-soft set on C where S is mapping given by $S:B\to 2^{U\times P}$ with the condition for each b∈B and ∃ a unique $(u,p_{b})\in U\times P$ such that $(u,p_{b})\in S(b),u\in U,p_{b}\in P$.

Example 2.6 Let $U=\{u_{1},u_{2},u_{3},u_{4}\}$ be a set of four different computers and $B=\{b_{1},b_{2},b_{3}\}$ be a set of consumers. The consumers provide their ratting values as P={0,1,2}. Then a 3-soft set $S:B\to 2^{U\times P}$ is expressed as follows;

$(S,B,3)=\left\{ \begin{array}{c} S(b_{1})=\{(u_{1},2),(u_{2},0),(u_{3},1),(u_{4},0)\}\\ S(b_{2})=\{(u_{1},1),(u_{2},2),(u_{3},1),(u_{4},2)\}\\ S(b_{1})=\{(u_{1},0),(u_{2},2),(u_{3},0),(u_{4},1)\}. \end{array}\right.$

Definition 2.7 [35] Let U be a collection of objects and C is a set of attributes. Let P={0,1,2,⋯,N−1} be a set of ordered grades, here N={2,3,⋯}, and B⊆C. A triple $\left(F_{q},B,N\right)$ is known as the q-rung orthopair fuzzy N-soft set (q-ROFNSS) on U where $F_{q}$ is mapping given by $F_{q}:B\to 2^{U\times P}\times q-ROFN$, such that for each u∈U and b∈B, ∃ $(u,p_{b})\in U\times P$ that is $p_{b}\in P$ and q−ROFN=〈ϕ(b),φ(b)〉, such that $\left(F_{q},B,N\right)$ can be defined as $F_{q}(b)=((u,p_{b}),\langle \phi (b),\varphi (b)\rangle )$. The level of the element of attribute denotes as $p_{b}$. The ϕ(b) and φ(b) represent the degrees of credible and non-credible information respectively, with the condition that $0\leq {\left(\phi (b)\right)}^{q}+{\left(\varphi (b)\right)}^{q}\leq 1$, ∀b∈B and q≥1.

In general a q-ROFNSS is denoted and defined by;

$(F_{q},B,N)=\left\{ \begin{array}{c} F_{q}(b_{1})=\left\{\begin{array}{c} ((u_{1},p_{11}),\langle \phi_{11},\varphi_{11}\rangle ),((u_{2},p_{21}),\langle \phi_{21},\varphi_{21}\rangle ),\\ \cdots ,((u_{m},p_{m1}),\langle \phi_{m1},\varphi_{m1}\rangle ) \end{array}\right\}\\ F_{q}(b_{2})=\left\{\begin{array}{c} ((u_{1},p_{12}),\langle \phi_{12},\varphi_{12}\rangle ),((u_{2},p_{22}),\langle \phi_{22},\varphi_{22}\rangle ),\\ \cdots ,((u_{m},p_{m2}),\langle \phi_{m2},\varphi_{m2}\rangle ) \end{array}\right\}\\ \;\;\vdots \\ F_{q}(b_{l})=\left\{\begin{array}{c} ((u_{1},p_{1l}),\langle \phi_{1l},\varphi_{1l}\rangle ),((u_{2},p_{2l}),\langle \phi_{2l},\varphi_{2l}\rangle ),\\ \cdots ,((u_{l},p_{ml}),\langle \phi_{ml},\varphi_{ml}\rangle ) \end{array}\right\}, \end{array}\right.$

where $p_{ji}\in P$ and $0\leq {\left(\phi_{ji}\right)}^{q}+{\left(\varphi_{ji}\right)}^{q}\leq 1$ ∀i=1,2,⋯,l,j=1,2,⋯,m. Furthermore, for any u∈U and b∈B, the pair τ=(p,〈ϕ,φ〉) is known as q-ROFN value (q-ROFNV) of an q-ROFNSS.

Remark 2.8 For any j∈{1,2,⋯,m}, $u_{j}\in U$, an N-parameterized q-rung orthopair fuzzy set (N-Pq-ROFS) is defined by;

$N-Pq-ROFS(u_{j})=\left\{\begin{array}{c} \left(p_{j1},\langle \phi_{j1},\varphi_{j1}\rangle ),(p_{j2},\langle \phi_{j2},\varphi_{j2}\rangle ),\cdots ,(p_{jl},\langle \phi_{jl},\varphi_{jl}\rangle \right) \end{array}\right\}$.

On aforementioned remark, we introduce operational rules for q-ROFNVs.

Definition 2.9 Let a set of q-ROFNVs $\left\{\tau_{1}=(p_{1},\langle \phi_{1},\varphi_{1}\rangle ),\tau_{2}=(p_{2},\langle \phi_{2},\varphi_{2}\rangle ),\cdots ,\tau_{m}=(p_{l},\langle \phi_{l},\varphi_{l}\rangle )\right\}$ and related weighted vector is given by $\left[\gamma_{1},\gamma_{2},\cdots ,\gamma_{l}\right]$. Then operational rule of q-ROFNVs is given by;

• $(\alpha_{i}p_{i}).\tau_{i}=\langle \sqrt[{q}]{\left(1-\prod_{i=1}^{l}{\left(1-{\left(\phi_{i}\right)}^{q}\right)}^{\left(\alpha_{i}p_{i}\right)}\right)},\prod_{i=1}^{l}{\left(\varphi_{i}\right)}^{\left(\alpha_{i}p_{i}\right)}\rangle$,
• ${\left(\tau_{i}\right)}^{\left(\alpha_{i}p_{i}\right)}=\langle \prod_{i=1}^{l}{\left(\phi_{i}\right)}^{\left(\alpha_{i}p_{i}\right)},\sqrt[{q}]{\left(1-\prod_{i=1}^{l}{\left(1-{\left(\varphi_{i}\right)}^{q}\right)}^{\left(\alpha_{i}p_{i}\right)}\right)}\rangle$.

To clarify, above results we investigated an example as in the following.

Example 2.10 Consider Example 2.6, where P={0,1,2}. Let b∈B, 0≤ϕ(b)≤1 and q=3 with $0\leq {\left(\phi (b)\right)}^{3}+{\left(\varphi (b)\right)}^{3}\leq 1$. The membership grade can be computed through;

• if $p_{b}=0$ then 0≤ϕ(b)<0.3,
• if $p_{b}=1$ then 0.3≤ϕ(b)<0.7,
• if $p_{b}=2$ then 0.7≤ϕ(b)<1.

The non-membership grade φ(b) can be any value representing non-credible information in [0,1] satisfying $0\leq {\left(\phi (b)\right)}^{3}+{\left(\varphi (b)\right)}^{3}\leq 1$. Then q-ROFNSS is given by;

$(F_{q},B,3)=\left\{ \begin{array}{c} F_{q}(b_{1})=\left\{\begin{array}{c} ((u_{1},2),\langle 0.70,0.60\rangle ),((u_{2},0),\langle 0.27,0.80\rangle ),\\ \left((u_{3},1),\langle 0.42,0.60\rangle ),((u_{4},0),\langle 0.22,0.50\rangle \right) \end{array}\right\}\\ F_{q}(b_{2})=\left\{\begin{array}{c} ((u_{1},1),\langle 0.43,0.70\rangle ),((u_{2},2),\langle 0.72,0.70\rangle ),\\ \left((u_{3},1),\langle 0.33,0.45\rangle ),(u_{4},2),\langle 0.74,0.80\rangle \right) \end{array}\right\}\\ F_{q}(b_{3})=\left\{\begin{array}{c} ((u_{1},0),\langle 0.26,0.56\rangle ),((u_{2},2),\langle 0.75,0.69\rangle ),\\ \left((u_{3},0),\langle 0.16,0.29\rangle ),((u_{4},1),\langle 0.39,0.52\rangle \right) \end{array}\right\}. \end{array}\right.$

Now, two different types of q-ROFNSS subsets are given in following two definitions.

Definition 2.11 Let P={0,1,2,⋯,N−1} be a set of ordered grades, here N={2,3,⋯}. Suppose two q-ROFNSS $T=(F_{q},B,N)$ and $Y=(G_{q},D,N)$ over U, where B⊆D, then we say that T is the subset of Y $\left((F_{q},B,N){⊑}_{1}(G_{q},D,N)\right)$ if and only if $\forall b_{j}\in B$ we have

$\left(p_{j1}\leq p_{j1}^{\prime },\phi_{j1}\leq \phi_{j1}^{\prime },\varphi_{j1}\geq \varphi_{j1}^{\prime }),(p_{j2}\leq p_{j2}^{\prime },\phi_{j2}\leq \phi_{j2}^{\prime },\varphi_{j2}\geq \varphi_{j2}^{\prime }),\cdots ,(p_{jl}\leq p_{jl}^{\prime },\phi_{jl}\leq \phi_{jl}^{\prime },\varphi_{jl}\geq \varphi_{jl}^{\prime }\right)$ where j=1,2,⋯,m.

Definition 2.12 Let P={0,1,2,⋯,N−1} be a set of ordered grades, here N={2,3,⋯}. Suppose two q-ROFNSS $T=(F_{q},B,N)$ and $Y=(G_{q},D,N)$ over U, where B⊆D, then we say that T is the subset of Y $\left((F_{q},B,N){⊑}_{2}(G_{q},D,N)\right)$ if and only if $\forall b_{j}\in B$ we have

$\left(p_{j1}\geq p_{j1}^{\prime },\phi_{j1}\leq \phi_{j1}^{\prime },\varphi_{j1}\geq \varphi_{j1}^{\prime }),(p_{j2}\geq p_{j2}^{\prime },\phi_{j2}\leq \phi_{j2}^{\prime },\varphi_{j2}\geq \varphi_{j2}^{\prime }),\cdots ,(p_{jl}\geq p_{jl}^{\prime },\phi_{jl}\leq \phi_{jl}^{\prime },\varphi_{jl}\geq \varphi_{jl}^{\prime }\right)$ where j=1,2,⋯,m.

Definition 2.13 [35] Let $\left(F_{q},B,N\right)$ be a q-ROFNSS over U. Then the weak complement $\left({F^{\prime }}_{q},B,N\right)$ of $\left(F_{q},B,N\right)$ is expressed as;

$({F^{\prime }}_{q},B,N)=((u,p_{b}),\langle \varphi b,\phi b\rangle ),(u,p_{b})\in U\times P$.

Definition 2.14 [35] Assume that $\left(F_{q},B,N\right)$ is a q-ROFNSS over U. The top weak complement $\left(F_{q}^{⊤},B,N\right)$ of $\left(F_{q},B,N\right)$ is expressed as;

$(F_{q}^{⊤},B,N)=\left\{ \begin{array}{c} ((u,N-1),\;\;\langle \varphi (b),\phi (b)\rangle ),\;\;if\;\;p_{b}<N-1,\\ ((u,0),\;\;\langle \varphi (b),\phi (b)\rangle ),\;\;if\;\;p_{b}=N-1. \end{array}\right.$

Definition 2.15 [35] Assume that $\left(F_{q},B,N\right)$ be a q-ROFNSS over U. A bottom weak complement $\left(F_{q}^{⊥},B,N\right)$ of $\left(F_{q},B,N\right)$ is expressed as;

$(F_{q}^{⊥},B,N)=\left\{ \begin{array}{c} ((u,0),\;\;\langle \varphi (b),\phi (b)\rangle ),\;\;if\;\;p_{b}>0,\\ ((u,N-1),\;\;\langle \varphi (b),\phi (b)\rangle ),\;\;if\;\;p_{b}=0. \end{array}\right.$

The aforementioned three distinct types of complements of q-ROFNVs are illustrated in the following example.

Example 2.16 Let us consider Example 2.10, where $\left(F_{q},B,3\right)$ is given for P={0,1,2}. Then weak, top weak, bottom weak complements are given by;

$({F^{\prime }}_{q},B,3)=\left\{ \begin{array}{c} F_{q}(b_{1})=\left\{\begin{array}{c} ((u_{1},2),\langle 0.60,0.70\rangle ),((u_{2},0),\langle 0.80,0.27\rangle ),\\ \left((u_{3},1),\langle 0.60,0.42\rangle ),((u_{4},0),\langle 0.50,0.22\rangle \right) \end{array}\right\}\\ F_{q}(b_{2})=\left\{\begin{array}{c} ((u_{1},1),\langle 0.70,0.43\rangle ),((u_{2},2),\langle 0.70,0.72\rangle ),\\ \left((u_{3},1),\langle 0.45,0.33\rangle ),(u_{4},2),\langle 0.80,0.74\rangle \right) \end{array}\right\}\\ F_{q}(b_{3})=\left\{\begin{array}{c} ((u_{1},0),\langle 0.56,0.26\rangle ),((u_{2},2),\langle 0.67,0.75\rangle ),\\ \left((u_{3},0),\langle 0.29,0.16\rangle ),((u_{4},1),\langle 0.52,0.39\rangle \right) \end{array}\right\}. \end{array}\right.$

$({F^{⊤}}_{q},B,3)=\left\{ \begin{array}{c} F_{q}(b_{1})=\left\{\begin{array}{c} ((u_{1},0),\langle 0.60,0.70\rangle ),((u_{2},2),\langle 0.80,0.27\rangle ),\\ \left((u_{3},2),\langle 0.60,0.42\rangle ),((u_{4},2),\langle 0.50,0.22\rangle \right) \end{array}\right\}\\ F_{q}(b_{2})=\left\{\begin{array}{c} ((u_{1},2),\langle 0.70,0.43\rangle ),((u_{2},0),\langle 0.70,0.72\rangle ),\\ \left((u_{3},2),\langle 0.45,0.33\rangle ),(u_{4},0),\langle 0.80,0.74\rangle \right) \end{array}\right\}\\ F_{q}(b_{3})=\left\{\begin{array}{c} ((u_{1},2),\langle 0.56,0.26\rangle ),((u_{2},0),\langle 0.67,0.75\rangle ),\\ \left((u_{3},2),\langle 0.29,0.16\rangle ),((u_{4},2),\langle 0.52,0.39\rangle \right) \end{array}\right\}. \end{array}\right.$

$({F^{⊥}}_{q},B,3)=\left\{ \begin{array}{c} F_{q}(b_{1})=\left\{\begin{array}{c} ((u_{1},0),\langle 0.60,0.70\rangle ),((u_{2},2),\langle 0.80,0.27\rangle ),\\ \left((u_{3},0),\langle 0.60,0.42\rangle ),((u_{4},2),\langle 0.50,0.22\rangle \right) \end{array}\right\}\\ F_{q}(b_{2})=\left\{\begin{array}{c} ((u_{1},0),\langle 0.70,0.43\rangle ),((u_{2},0),\langle 0.70,0.72\rangle ),\\ \left((u_{3},0),\langle 0.45,0.33\rangle ),(u_{4},0),\langle 0.80,0.74\rangle \right) \end{array}\right\}\\ F_{q}(b_{3})=\left\{\begin{array}{c} ((u_{1},2),\langle 0.56,0.26\rangle ),((u_{2},0),\langle 0.67,0.75\rangle ),\\ \left((u_{3},2),\langle 0.29,0.16\rangle ),((u_{4},2),\langle 0.52,0.39\rangle \right) \end{array}\right\}. \end{array}\right.$

**Group-based generalized q-rung orthopair fuzzy N-soft sets (GGq-ROFNSS)**

In this part of article, important notion of GGq-ROFNSS is investigated and underlying operations are elaborated.

Definition 2.17 Let U be a collection of objects and $B=\{c_{1},c_{2},\cdots ,c_{l}\}\subseteq C$ be a set of attributes. Let P={0,1,2,⋯,N−1} be a set of ordered grades, here N={2,3,⋯}. A quadruple $T_{K}=(F_{q},B,N,K)$ is known as the group-based generalized q-rung orthopair fuzzy N-soft set (GGq-ROFNSS) on U if and only if $\left(F_{q},B,N\right)$ is the elementary q-ROFNSS (Eq-ROFNSS) and $K=\{K_{M_{1}},K_{M_{2}},\cdots ,K_{M_{n}}\}$ is the set of N-Pq-ROFSs for vital evaluations of moderators $M_{r^{\prime }}(r^{\prime }=1,2,\cdots ,n)$ over set of attributes.

Another view of the above definition of GGq-ROFNSS is indicated in the following notion.

Definition 2.18 Let $T_{K}=(F_{q},B,N,K)$ be the GGq-ROFNSS over U and it is shown in Table 1, where the orange part is representing Eq-ROFNSS while gray part is representing set of N-Pq-ROFSs for vital evaluations of moderators $M_{r^{\prime }}(r^{\prime }=1,2,\cdots ,n)$ over set of criteria. Take r=0,1,2,⋯,n such that GGq-ROFNSS $T_{K}$ is represented as;

**Table 1:**
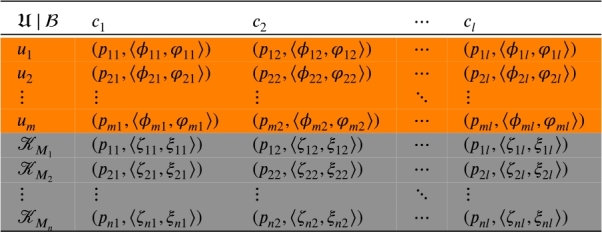
The tabular representation of the GGq-ROFNSS $T_{K}=(F_{q},B,N,K)$.

${\overset{˜}{\tau }}_{ji}^{r}=(p_{ji}^{r},\langle \phi_{ji}^{r},\varphi_{ji}^{r}\rangle )=\left\{ \begin{array}{c} (p_{ji},\langle \phi_{ji},\varphi_{ji}\rangle ),\;\;if\;\;r=0.\\ (p_{r^{\prime }i},\langle \zeta_{r^{\prime }i},\xi_{r^{\prime }i}\rangle ),\;\;if\;\;r>0. \end{array}\right.$, where j=1,2,⋯,m and i=1,2,⋯,l.

In other words, ${\overset{˜}{\tau }}_{ji}^{0}=(p_{ji}^{0},\langle \phi_{ji}^{0},\varphi_{ji}^{0}\rangle )=(p_{ji},\langle \phi_{ji},\varphi_{ji}\rangle )$ represent q-ROFNVs in Eq-ROFNSS (see orange part of Table 1) and ${\overset{˜}{\tau }}_{ji}^{r^{\prime }}=(p_{ji}^{r^{\prime }},\langle \phi_{ji}^{r^{\prime }},\varphi_{ji}^{r^{\prime }}\rangle )=(p_{r^{\prime }i},\langle \zeta_{r^{\prime }i},\xi_{r^{\prime }i}\rangle )$ represents q-ROFNVs in $r^{\prime }$ number of N-Pq-ROFSs (see gray part of Table 1).

Definition 2.18 is the q-ROFNS numbers form of Definition 2.17, it is useful to define operations on GGq-ROFNSSs.

## 3 Operations on GGq-ROFNSS

In this part of study, we express elementary operations on GGq-ROFNSSs.

Definition 3.1 Let P={0,1,2,⋯,N−1} be a set of ordered grades, where N={2,3,⋯}. Let two GGq-ROFNSSs $T_{K}=(F_{q},B,N,K)$ and $Y_{E}=(G_{q},D,N,E)$ over U, where B⊆D, $K=\{K_{M_{1}},K_{M_{2}},\cdots ,K_{M_{n}}\}$, $E=\{E_{M_{1}},E_{M_{2}},\cdots ,E_{M_{n}}\}$ and

$M_{1},M_{2},\cdots ,M_{n}$ are “n” numbers of senior moderators. Then K is the subset of E ($K\subseteq_{1}E$) if and only if for $b_{i}\in B$ we have;

$(p_{1i}\leq p_{1i}^{\prime },\zeta_{K_{M_{1}}}(b_{i})\leq \zeta_{E_{M_{1}}}(b_{i}),\xi_{K_{M_{1}}}(b_{i})\geq \xi_{E_{M_{1}}}(b_{i})),(p_{2i}\leq p_{2i}^{\prime },\zeta_{K_{M_{2}}}(b_{i})\leq \zeta_{E_{M_{2}}}(b_{i}),\xi_{K_{M_{2}}}(b_{i})\geq \xi_{E_{M_{2}}}(b_{i})),\cdots$,

$(p_{in}\leq p_{in}^{\prime },\zeta_{K_{M_{1}}}(b_{i})\leq \zeta_{E_{M_{1}}}(b_{i}),\xi_{K_{M_{1}}}(b_{i})\geq \xi_{E_{M_{1}}}(b_{i})),\forall i=1,2,\cdots ,l$.

Definition 3.2 Let P={0,1,2,⋯,N−1} be a set of ordered grades, where N={2,3,⋯}. Let two GGq-ROFNSSs $T_{K}=(F_{q},B,N,K)$ and $Y_{E}=(G_{q},D,N,E)$ over U, where B⊆D, $K=\{K_{M_{1}},K_{M_{2}},\cdots ,K_{M_{n}}\}$ and $E=\{E_{M_{1}},E_{M_{2}},\cdots ,E_{M_{n}}\}$ and

$M_{1},M_{2},\cdots ,M_{n}$ are “n” numbers of senior moderators. Then K is the subset of E ($K\subseteq_{2}E$) if and only if for $b_{i}\in B$ we have;

$(p_{1i}\geq p_{1i}^{\prime },\zeta_{K_{M_{1}}}(b_{i})\leq \zeta_{E_{M_{1}}}(b_{i}),\xi_{K_{M_{1}}}(b_{i})\geq \xi_{E_{M_{1}}}(b_{i})),(p_{2i}\geq p_{2i}^{\prime },\zeta_{K_{M_{2}}}(b_{i})\leq \zeta_{E_{M_{2}}}(b_{i}),\xi_{K_{M_{2}}}(b_{i})\geq \xi_{E_{M_{2}}}(b_{i})),\cdots$,

$(p_{in}\geq p_{in}^{\prime },\zeta_{K_{M_{1}}}(b_{i})\leq \zeta_{E_{M_{1}}}(b_{i}),\xi_{K_{M1}}(b_{i})\geq \xi_{E_{M_{1}}}(b_{i})),\forall i=1,2,\cdots ,l$.

Definition 3.3 Let P={0,1,2,⋯,N−1} be a set of ordered grades, where N={2,3,⋯}. Let two GGq-ROFNSSs $T_{K}=(F_{q},B,N,K)$ and $Y_{E}=(G_{q},D,N,E)$ over U, where B⊆D. Then $T_{K}$ is a group based generalized q-rung orthopair fuzzy N-soft set $Z_{1}$-subset of $Y_{E}$ represented as $T_{K}{\text{⊆}}_{1}Y_{E}$, then

• $\left(F_{q},B,N){⊑}_{1}(G_{q},D,N\right)$,
• $K\subseteq_{1}E$.

Definition 3.4 Let P={0,1,2,⋯,N−1} be a set of ordered grades, where N={2,3,⋯}. Let two GGq-ROFNSSs $T_{K}=(F_{q},B,N,K)$ and $Y_{E}=(G_{q},D,N,E)$ over U, where B⊆D. Then $T_{K}$ is a group based generalized q-rung orthopair fuzzy N-soft set $Z_{2}$-subset of $Y_{E}$ represented as $T_{K}{\text{⊆}}_{2}Y_{E}$, then

• $\left(F_{q},B,N){⊑}_{2}(G_{q},D,N\right)$,
• $K\subseteq_{2}E$.

Definition 3.5 Let $T_{K}=(F_{q},B,N,K)$ be a GGq-ROFNSS over U as shown in Table 1. The weak complement $\left({F^{\prime }}_{q},B,N,K\right)$ of $\left(F_{q},B,N,K\right)$ is expressed as;

${\left({\overset{˜}{\tau }}^{c}\right)}_{ji}^{r}=(p_{ji}^{r},\langle \varphi_{ji}^{r},\phi_{ji}^{r}\rangle )=\left\{ \begin{array}{c} (p_{ji},\langle \varphi_{ji},\phi_{ji}\rangle ),\;\;if\;\;r=0,\\ (p_{r^{\prime }i},\langle \xi_{r^{\prime }i},\zeta_{r^{\prime }i}\rangle ),\;\;if\;\;r>0. \end{array}\right.$

For different settings of ratting values we define the following two notions.

Definition 3.6 Let $\left(F_{q},B,N,K\right)$ be a GGq-ROFNSS over U as shown in Table 1. The top weak complement $\left(F_{q}^{⊤},B,N,K\right)$ of $\left(F_{q},B,N,K\right)$ is expressed as;

${\left({\overset{˜}{\tau }}^{⊤}\right)}_{ji}^{r}=\left\{ \begin{array}{c} (N-1,\langle \varphi_{ji}^{r},\phi_{ji}^{r}\rangle ),\;\;if\;\;p_{ji}<N-1,\\ (0,\langle \varphi_{ji}^{r},\phi_{ji}^{r}\rangle ),\;\;if\;\;p_{ji}=N-1^{\prime } \end{array}\right.$ where

$(N-1,\langle \varphi_{ji}^{r},\phi_{ji}^{r}\rangle )=\left\{ \begin{array}{c} (N-1,\langle \varphi_{ji},\phi_{ji}\rangle ),\;\;if\;\;r=0,\\ (N-1,\langle \xi_{r^{\prime }i},\zeta_{r^{\prime }i}\rangle ),\;\;if\;\;r>0, \end{array}\right.$ and

$(0,\langle \varphi_{ji}^{r},\phi_{ji}^{r}\rangle )=\left\{ \begin{array}{c} (0,\langle \varphi_{ji},\phi_{ji}\rangle ),\;\;if\;\;r=0,\\ (0,\langle \xi_{r^{\prime }i},\zeta_{r^{\prime }i}\rangle ),\;\;if\;\;r>0. \end{array}\right.$

Definition 3.7 Let $\left(F_{q},B,N,K\right)$ be a GGq-ROFNSS over U as shown in Table 1. The bottom weak complement $\left(F_{q}^{⊥},B,N,K\right)$ of $\left(F_{q},B,N,K\right)$ is expressed as;

${\left({\overset{˜}{\tau }}^{⊥}\right)}_{ji}^{r}=\left\{ \begin{array}{c} (0,\langle \varphi_{ji}^{r},\phi_{ji}^{r}\rangle ),\;\;if\;\;p_{ji}>0,\\ (N-1,\langle \varphi_{ji}^{r},\phi_{ji}^{r}\rangle ),\;\;if\;\;p_{ji}=0^{\prime } \end{array}\right.$ where

$(0,\langle \varphi_{ji}^{r},\phi_{ji}^{r}\rangle )=\left\{ \begin{array}{c} (0,\langle \varphi_{ji},\phi_{ji}\rangle ),\;\;if\;\;r=0,\\ (0,\langle \xi_{r^{\prime }i},\zeta_{r^{\prime }i}\rangle ),\;\;if\;\;r>0, \end{array}\right.$ and

$(N-1,\langle \varphi_{ji}^{r},\phi_{ji}^{r}\rangle )=\left\{ \begin{array}{c} (N-1,\langle \varphi_{ji},\phi_{ji}\rangle ),\;\;if\;\;r=0,\\ (N-1,\langle \xi_{r^{\prime }i},\zeta_{r^{\prime }i}\rangle ),\;\;if\;\;r>0. \end{array}\right.$

Example 1 Consider a set of four different solar cells

$U=\left\{\begin{array}{c} u_{1}=Thin\;\;film\;\;solar\;\;cells,u_{2}=Monocrystalline\;\;cells,\\ u_{3}=Polycrystalline\;\;cells,u_{4}=Crystalline\;\;silicon\;\;cells \end{array}\right\}$.

An industry H who manufactures biscuits and snacks, wants to produce electricity by using solar cells. Industry plans to choose suitable solar cell from U in term of cost and reliability. A set of three parameters is considered by industry $B=\{b_{1},b_{2},b_{3}\}\subset C$, where $b_{i}(1,2,3)$, respectively, stand for, cost of solar cell per square meter, power efficiency and environmental free (led free)and N={0,1,2,3,4,5}. A team of experts from industry provides the assessments in the form of Eq-ROFNSS as follows;

$F_{q}(c_{1})=\{\frac{u_{1}}{\left(2,\langle 0.10,0.12\rangle \right)},\frac{u_{2}}{\left(1,\langle 0.13,0.23\rangle \right)},\frac{u_{3}}{\left(5,\langle 0.23,0.28\rangle \right)},\frac{u_{4}}{\left(4,\langle 0.21,0.41\rangle \right)}\}$,

$F_{q}(c_{2})=\{\frac{u_{1}}{\left(3,\langle 0.25,0.35\rangle \right)},\frac{u_{2}}{\left(2,\langle 0.35,0.45\rangle \right)},\frac{u_{3}}{\left(4,\langle 0.22,0.28\rangle \right)},\frac{u_{4}}{\left(0,\langle 0.36,0.44\rangle \right)}\}$,

$F_{q}(c_{3})=\{\frac{u_{1}}{\left(2,\langle 0.31,0.37\rangle \right)},\frac{u_{2}}{\left(3,\langle 0.30,0.39\rangle \right)},\frac{u_{3}}{\left(2,\langle 0.29,0.33\rangle \right)},\frac{u_{4}}{\left(1,\langle 0.19,0.31\rangle \right)}\}$.

Consider three solar power experts ${⋎}_{1},{⋎}_{2}$ and ${⋎}_{3}$ as moderators who provide assessments as rating values on each parameter in C. In other word moderators provides their assessments (as PGGq-ROFNSs) to surmise data in Eq-ROFNSS. Taking q=4, PGGq-ROFNSs of ${⋎}_{1},{⋎}_{2}$ and ${⋎}_{3}$ are $K_{{⋎}_{1}},K_{{⋎}_{2}}$ and $K_{{⋎}_{3}}$ respectively, and given by;

$K=\left\{ \begin{array}{c} K_{{⋎}_{1}}=\{\frac{b_{1}}{\left(2,\langle 0.09,0.71\rangle \right)},\frac{b_{2}}{\left(1,\langle 0.20,0.30\rangle \right)},\frac{b_{3}}{\left(3,\langle 0.24,0.28\rangle \right)}\},\\ K_{{⋎}_{2}}=\{\frac{b_{1}}{\left(4,\langle 0.05,0.65\rangle \right)},\frac{b_{2}}{\left(2,\langle 0.30,0.44\rangle \right)},\frac{b_{3}}{\left(4,\langle 0.12,0.16\rangle \right)}\},\\ K_{{⋎}_{3}}=\{\frac{b_{1}}{\left(2,\langle 0.13,0.37\rangle \right)},\frac{b_{2}}{\left(2,\langle 0.42,0.50\rangle \right)},\frac{b_{3}}{\left(3,\langle 0.27,0.44\rangle \right)}\} \end{array}\right.$

Therefore the GGq-ROF6SSs $T_{K}=(F_{q},B,6,K)$ can be expressed as in Table 2. The weak complement, top weak complement and bottom weak complement of GGq-ROF6SSs in Table 2 are depicted in Table 3, Table 4, Table 5, respectively.

**Table 2:** The tabular representation of the GGq-ROFNSS $T_{K}=(F_{q},B,6,K)$.

U|B	*b*_1_	*b*_2_	*b*_3_
*u*_1_	(2,〈0.10,0.12〉)	(3,〈0.25,0.35〉)	(2,〈0.31,0.37〉)
*u*_2_	(1,〈0.13,0.23〉)	(2,〈0.35,0.45〉)	(3,〈0.30,0.39〉)
*u*_3_	(5,〈0.23,0.28〉)	(4,〈0.26,0.28〉)	(2,〈0.29,0.33〉)
*u*_4_	(4,〈0.21,0.41〉)	(0,〈0.36,0.44〉)	(1,〈0.19,0.31〉)

$K_{{⋎}_{1}}$	(3,〈0.09,0.71〉)	(4,〈0.05,0.65〉)	(2,〈0.13,0.37〉)
$K_{{⋎}_{2}}$	(1,〈0.20,0.30〉)	(3,〈0.30,0.40〉)	(2,〈0.42,0.50〉)
$K_{{⋎}_{3}}$	(3,〈0.24,0.28〉)	(4,〈0.12,0.16〉)	(3,〈0.27,0.44〉)

**Table 3:** Weak complement $\left({F^{\prime }}_{q},B,6,K\right)$ of $\left(F_{q},B,6,K\right)$.

U|B	*b*_1_	*b*_2_	*b*_3_
*u*_1_	(2,〈0.12,0.10〉)	(3,〈0.35,0.25〉)	(2,〈0.37,0.31〉)
*u*_2_	(1,〈0.23,0.13〉)	(2,〈0.45,0.35〉)	(3,〈0.39,0.30〉)
*u*_3_	(5,〈0.28,0.23〉)	(4,〈0.28,0.26〉)	(2,〈0.33,0.29〉)
*u*_4_	(4,〈0.41,0.21〉)	(0,〈0.44,0.36〉)	(1,〈0.31,0.19〉)

$K_{{⋎}_{1}}$	(3,〈0.71,0.09〉)	(4,〈0.65,0.05〉)	(2,〈0.37,0.31〉)
$K_{{⋎}_{2}}$	(1,〈0.30,0.20〉)	(3,〈0.40,0.30〉)	(2,〈0.50,0.42〉)
$K_{{⋎}_{3}}$	(3,〈0.28,0.24〉)	(4,〈0.16,0.12〉)	(3,〈0.44,0.27〉)

**Table 4:** Top weak complement $\left({F^{⊤}}_{q},B,6,K\right)$ of $\left(F_{q},B,6,K\right)$.

U|B	*b*_1_	*b*_2_	*b*_3_
*u*_1_	(5,〈0.12,0.10〉)	(5,〈0.35,0.25〉)	(5,〈0.37,0.31〉)
*u*_2_	(5,〈0.23,0.13〉)	(5,〈0.45,0.35〉)	(5,〈0.39,0.30〉)
*u*_3_	(5,〈0.28,0.23〉)	(5,〈0.28,0.26〉)	(5,〈0.33,0.29〉)
*u*_4_	(5,〈0.41,0.21〉)	(5,〈0.44,0.36〉)	(5,〈0.31,0.19〉)

$K_{{⋎}_{1}}$	(5,〈0.71,0.09〉)	(5,〈0.65,0.05〉)	(5,〈0.37,0.31〉)
$K_{{⋎}_{2}}$	(5,〈0.30,0.20〉)	(5,〈0.40,0.30〉)	(5,〈0.50,0.42〉)
$K_{{⋎}_{3}}$	(5,〈0.28,0.24〉)	(5,〈0.16,0.12〉)	(5,〈0.44,0.27〉)

**Table 5:** Bottom weak complement $\left({F^{⊥}}_{q},B,6,K\right)$ of $\left(F_{q},B,6,K\right)$.

U|B	*b*_1_	*b*_2_	*b*_3_
*u*_1_	(0,〈0.12,0.10〉)	(0,〈0.35,0.25〉)	(0,〈0.37,0.31〉)
*u*_2_	(0,〈0.23,0.13〉)	(0,〈0.45,0.35〉)	(0,〈0.39,0.30〉)
*u*_3_	(0,〈0.28,0.23〉)	(0,〈0.28,0.26〉)	(0,〈0.33,0.29〉)
*u*_4_	(0,〈0.41,0.21〉)	(0,〈0.44,0.36〉)	(0,〈0.31,0.19〉)

$K_{{⋎}_{1}}$	(0,〈0.71,0.09〉)	(0,〈0.65,0.05〉)	(0,〈0.37,0.31〉)
$K_{{⋎}_{2}}$	(0,〈0.30,0.20〉)	(0,〈0.40,0.30〉)	(0,〈0.50,0.42〉)
$K_{{⋎}_{3}}$	(0,〈0.28,0.24〉)	(0,〈0.16,0.12〉)	(0,〈0.44,0.27〉)

**Group-based generalized q-rung orthopair fuzzy N-soft weighted averaging (GGq-ROFNSWA) operators**

Although the GGq-ROFNSS is a superior structure since it incorporates several evaluations, a better aggregating mechanism is also necessary in addition to a reliable assessment model. In this section, GGq-ROFNSWA operators are introduced.

Definition 3.8 Let $T_{K}=(F_{q},B,N,K)$ be a GGq-ROFNSS over U as shown in Table 1. Let ${\left[\beta_{1},\beta_{2},\cdots ,\beta_{l}\right]}^{T}$ be a weighted vector over set of parameters such that $\beta_{i}>0$ and $\sum_{i=1}^{l}\beta_{i}=1$. Let weighted vector ${\left[\alpha_{0},\alpha_{1},\alpha_{2},\cdots ,\alpha_{n}\right]}^{T}$, such that $\alpha_{r}>0$ and $\sum_{r=0}^{n}\alpha_{r}=1$, where $\alpha_{1},\alpha_{2},\cdots ,\alpha_{n}$ are the weights over moderators, and $\alpha_{0}$ is the weight for the entire data set in Eq-ROFNSS (which is shown in orange part of Table 1). Let $\left[\gamma_{1},\gamma_{2},\cdots ,\gamma_{m}\right]$ be the set of essential weights of alternatives, with condition $\gamma_{m}>0$ and $\sum_{j=1}^{m}\gamma_{m}=1$. Let r=0,1,2,⋯,n, then the GGq-ROFNSWA operators on the GGq-ROFNSS are given as follows.

$\Lambda =WA^{GGq-ROFNSS}={⊕}_{j=1}^{m}\gamma_{j}({⊕}_{i=1}^{l}\beta_{i}({⊕}_{r=0}^{n}(\alpha_{r}p_{ji}^{r}).{\overset{˜}{\tau }}_{ji}^{r}))$ where $WA^{GGq-ROFNSS}$ denoted GGq-ROFNSWA operators on GGq-ROFNSS.

Theorem 3.9 *Suppose that* ${\overset{˜}{\tau }}_{ij}^{r}=\langle {\left(\phi \right)}_{ji}^{r},{\left(\varphi \right)}_{ji}^{r}\rangle$ *, be a GGq-ROFNSVs. So* (i=1,2,3,⋯,l,j=1,2,⋯,m) *and* r=0,1,2,⋯,n *. where GGq-ROFNSWA operator is given below as;*

$WA^{GGq-ROFNSS}={⊕}_{j=1}^{m}\gamma_{j}({⊕}_{i=1}^{l}\beta_{i}({⊕}_{r=0}^{n}(\alpha_{r}p_{ji}^{r}){\overset{˜}{\tau }}_{ji}^{r}))=$

$\left(\begin{array}{c} \langle \sqrt[{q}]{1-\prod_{j=1}^{m}{\left(\prod_{i=1}^{l}{\left(\prod_{r=0}^{n}{\left(1-{\left({\left(\phi \right)}_{ji}^{r}\right)}^{q}\right)}^{\left(p_{ji}^{r}\alpha_{r}\right)}\right)}^{\beta_{i}}\right)}^{\gamma_{j}}},\prod_{j=1}^{m}{\left(\prod_{i=1}^{l}{\left(\prod_{r=1}^{n}{\left({\left(\varphi \right)}_{ji}^{r}\right)}^{\left(p_{ji}^{r}\alpha_{r}\right)}\right)}^{\beta_{i}}\right)}^{\gamma_{j}}\rangle \end{array}\right)$ *.*

Proof The above result can be proved by using mathematical induction.

Let m=2,l=2 and n=1. Then

$WA^{GGq-ROFNSS}={⊕}_{j=1}^{2}\gamma_{r}({⊕}_{i=1}^{2}\beta_{i}({⊕}_{r=0}^{1}(p_{ji}^{r}\alpha_{r}){\overset{˜}{\tau }}_{ji}^{r}))$

$=\gamma_{1}({⊕}_{i=1}^{2}\beta_{i}({⊕}_{r=0}^{1}(p_{1i}^{r}\alpha_{r}){\overset{˜}{\tau }}_{1i}^{r}))⊕\gamma_{2}({⊕}_{i=1}^{2}\beta_{i}({⊕}_{r=0}^{1}(p_{2i}^{r}\alpha_{r}){\overset{˜}{\tau }}_{2i}^{r}))$

$=(\gamma_{1}((\beta_{1}(((p_{11}^{0}\alpha_{0}){\overset{˜}{\tau }}_{11}^{0})⊕((p_{11}^{1}\alpha_{1}){\overset{˜}{\tau }}_{11}^{1})))⊕(\beta_{2}(((p_{12}^{0}\alpha_{0}){\overset{˜}{\tau }}_{12}^{0})⊕((p_{12}^{1}\alpha_{1}){\overset{˜}{\tau }}_{12}^{1})))))⊕(\gamma_{2}((\beta_{1}(((p_{21}^{0}\alpha_{0}){\overset{˜}{\tau }}_{21}^{0})⊕((p_{21}^{1}\alpha_{1}){\overset{˜}{\tau }}_{21}^{1})))⊕(\beta_{2}(((p_{22}^{0}\alpha_{0}){\overset{˜}{\tau }}_{22}^{0})$

$⊕((p_{22}^{1}\alpha_{1}){\overset{˜}{\tau }}_{22}^{1}))))))$

= $\left(\begin{array}{c} \gamma_{1}\left(\begin{array}{c} \left(\begin{array}{c} \beta_{1}\left(\begin{array}{c} (\langle \sqrt[{q}]{1-{\left(1-{\left({\left(\phi \right)}_{11}^{0}\right)}^{q}\right)}^{\left(p_{11}^{0}\alpha_{0}\right)}},{\left({\left(\varphi \right)}_{11}^{0}\right)}^{\left(p_{11}^{0}\alpha_{0}\right)}\rangle )⊕\\ \left(\langle \sqrt[{q}]{1-{\left(1-{\left({\left(\phi \right)}_{11}^{1}\right)}^{q}\right)}^{\left(p_{11}^{1}\alpha_{1}\right)}},{\left({\left(\varphi \right)}_{11}^{1}\right)}^{\left(p_{11}^{1}\alpha_{1}\right)}\rangle \right) \end{array}\right) \end{array}\right)⊕\\ \left(\begin{array}{c} \beta_{2}\left(\begin{array}{c} (\langle \sqrt[{q}]{1-{\left(1-{\left({\left(\phi \right)}_{12}^{0}\right)}^{q}\right)}^{\left(p_{12}^{0}\alpha_{0}\right)}},{\left({\left(\varphi \right)}_{12}^{0}\right)}^{\left(p_{12}^{0}\alpha_{0}\right)}\rangle )⊕\\ \left(\langle \sqrt[{q}]{1-{\left(1-{\left({\left(\phi \right)}_{12}^{1}\right)}^{q}\right)}^{\left(p_{12}^{1}\alpha_{1}\right)}},{\left({\left(\varphi \right)}_{12}^{1}\right)}^{\left(p_{12}^{1}\alpha_{1}\right)}\rangle \right) \end{array}\right) \end{array}\right) \end{array}\right)⊕\\ \gamma_{2}\left(\begin{array}{c} \left(\begin{array}{c} \beta_{1}\left(\begin{array}{c} (\langle \sqrt[{q}]{1-{\left(1-{\left({\left(\phi \right)}_{21}^{0}\right)}^{q}\right)}^{\alpha_{0}}},{\left({\left(\varphi \right)}_{21}^{0}\right)}^{p_{21}^{0}\alpha_{0}}\rangle )⊕\\ \left(\langle \sqrt[{q}]{1-{\left(1-{\left({\left(\phi \right)}_{21}^{1}\right)}^{q}\right)}^{\left(p_{21}^{1}\alpha_{1}\right)}},{\left({\left(\varphi \right)}_{21}^{1}\right)}^{\left(p_{21}^{1}\alpha_{1}\right)}\rangle \right) \end{array}\right) \end{array}\right)⊕\\ \left(\begin{array}{c} \beta_{2}\left(\begin{array}{c} (\langle \sqrt[{q}]{1-{\left(1-{\left({\left(\phi \right)}_{22}^{0}\right)}^{q}\right)}^{\left(p_{22}^{0}\alpha_{0}\right)}},{\left({\left(\varphi \right)}_{22}^{0}\right)}^{\left(p_{22}^{0}\alpha_{0}\right)}\rangle )⊕\\ \left(\langle \sqrt[{q}]{1-{\left(1-{\left({\left(\phi \right)}_{22}^{1}\right)}^{q}\right)}^{\left(p_{22}^{1}\alpha_{1}\right)}},{\left({\left(\varphi \right)}_{22}^{1}\right)}^{\left(p_{22}^{1}\alpha_{1}\right)}\rangle \right) \end{array}\right) \end{array}\right) \end{array}\right) \end{array}\right)$

= $\left(\begin{array}{c} \gamma_{1}\left(\begin{array}{c} \left(\begin{array}{c} \beta_{1}\left(\begin{array}{c} \langle \sqrt[{q}]{1-\prod_{r=0}^{1}{\left(1-{\left({\left(\phi \right)}_{11}^{r}\right)}^{q}\right)}^{\left(p_{11}^{r}\alpha_{r}\right)}},{\left(\prod_{r=0}^{1}{\left(\varphi \right)}_{11}^{r}\right)}^{\left(p_{11}^{r}\alpha_{r}\right)}\rangle \end{array}\right) \end{array}\right)⊕\\ \left(\begin{array}{c} \beta_{2}\left(\begin{array}{c} \langle \sqrt[{q}]{1-\prod_{r=0}^{1}{\left(1-{\left({\left(\phi \right)}_{12}^{r}\right)}^{q}\right)}^{\left(p_{12}^{r}\alpha_{r}\right)}},{\left(\prod_{r=0}^{1}{\left(\varphi \right)}_{12}^{r}\right)}^{\left(p_{12}^{r}\alpha_{r}\right)}\rangle \end{array}\right) \end{array}\right) \end{array}\right)⊕\\ \gamma_{2}\left(\begin{array}{c} \left(\begin{array}{c} \beta_{1}\left(\begin{array}{c} \langle \sqrt[{q}]{1-\prod_{r=0}^{1}{\left(1-{\left({\left(\phi \right)}_{21}^{r}\right)}^{q}\right)}^{\left(p_{21}^{r}\alpha_{r}\right)}},{\left(\prod_{r=0}^{1}{\left(\varphi \right)}_{21}^{r}\right)}^{\left(p_{21}^{r}\alpha_{r}\right)}\rangle \end{array}\right) \end{array}\right)⊕\\ \left(\begin{array}{c} \beta_{2}\left(\begin{array}{c} \langle \sqrt[{q}]{1-\prod_{r=0}^{1}{\left(1-{\left({\left(\phi \right)}_{22}^{r}\right)}^{q}\right)}^{\left(p_{22}^{r}\alpha_{r}\right)}},{\left(\prod_{r=0}^{1}{\left(\varphi \right)}_{22}^{r}\right)}^{\left(p_{22}^{r}\alpha_{r}\right)}\rangle \end{array}\right) \end{array}\right) \end{array}\right) \end{array}\right)$

= $\left(\begin{array}{c} \gamma_{1}\left(\begin{array}{c} \langle \sqrt[{q}]{1-\prod_{i=1}^{2}{\left(\prod_{r=0}^{1}{\left(1-{\left({\left(\phi \right)}_{1i}^{r}\right)}^{q}\right)}^{\left(p_{1i}^{r}\alpha_{r}\right)}\right)}^{\beta_{i}}},{\left(\prod_{i=1}^{2}{\left(\prod_{r=0}^{1}{\left(\varphi \right)}_{1i}^{r}\right)}^{\left(p_{1i}^{r}\alpha_{r}\right)}\right)}^{\beta_{i}})\rangle \end{array}\right)⊕\\ \gamma_{2}\left(\begin{array}{c} {\langle \sqrt[{q}]{1-\prod_{i=1}^{2}{\left(\prod_{r=0}^{1}{\left(1-{\left({\left(\phi \right)}_{2i}^{r}\right)}^{q}\right)}^{\left(p_{2i}^{r}\alpha_{r}\right)}\right)}^{\beta_{i}}},\prod_{i=1}^{2}{\left(\prod_{r=0}^{1}{\left(\varphi \right)}_{2i}^{r}\right)}^{\left(p_{2i}^{r}\alpha_{r}\right)})}^{\beta_{i}}\rangle \end{array}\right) \end{array}\right)$

= $\langle \sqrt[{q}]{1-\prod_{j=1}^{2}{\left(\prod_{i=1}^{2}{\left(\prod_{r=0}^{1}{\left(1-{\left({\left(\phi \right)}_{ji}^{r}\right)}^{q}\right)}^{\left(p_{ji}^{r}\alpha_{r}\right)}\right)}^{\beta_{i}}\right)}^{\gamma_{j}}},\prod_{j=1}^{2}{\left(\prod_{i=1}^{2}{\left(\prod_{r=0}^{1}{\left({\left(\varphi \right)}_{ji}^{r}\right)}^{\left(p_{ji}^{r}\alpha_{r}\right)}\right)}^{\beta_{i}}\right)}^{\gamma_{j}}\rangle$

Hence the result is true for m=2,l=2 and n=1. Now we take $m=m_{1},l=l_{1}$ and $n=n_{1}$.

= $\langle \sqrt[{q}]{1-\prod_{j=1}^{m_{1}}{\left(\prod_{i=1}^{l_{1}}{\left(\prod_{r=0}^{n_{1}}{\left(1-{\left({\left(\phi \right)}_{ji}^{r}\right)}^{q}\right)}^{\left(p_{ji}^{r}\alpha_{r}\right)}\right)}^{\beta_{i}}\right)}^{\gamma_{j}}},\prod_{j=1}^{m_{1}}{\left(\prod_{i=1}^{l_{1}}{\left(\prod_{r=0}^{n_{1}}{\left({\left(\varphi \right)}_{ji}^{r}\right)}^{\left(p_{ji}^{r}\alpha_{r}\right)}\right)}^{\beta_{i}}\right)}^{\gamma_{j}}\rangle$

Further we take as $m=m_{1}+1,l=l_{1}+1$ and $n=n_{1}+1$

$WA^{GGq-ROFNS}={⊕}_{j=1}^{\left(m_{1}+1\right)}\gamma_{j}({⊕}_{i=1}^{\left(l_{1}+1\right)}\beta_{i}({⊕}_{r=0}^{\left(n_{1}+1\right)}(p_{ji}^{r}\alpha_{r}){\overset{˜}{\tau }}_{ji}^{r}))$

= $\left({⊕}_{j=1}^{m_{1}}\gamma_{j}({⊕}_{i=1}^{l_{1}}\beta_{i}({⊕}_{r=0}^{n_{1}}(p_{ji}^{r}\alpha_{r}){\overset{˜}{\tau }}_{ji}^{r})))⊕(\gamma_{m_{1}+1}(\beta_{l_{1}+1}((p_{\left(m_{1}+1)(l_{1}+1\right)}^{n_{1}+1})(\alpha_{n_{1}+1})){\overset{˜}{\tau }}_{\left(l_{1}+1)(m_{1}+1\right)}^{n_{1}+1})\right)$

= $\left(\begin{array}{c} \langle \sqrt[{q}]{1-\prod_{j=1}^{m_{1}}{\left(\prod_{i=1}^{l_{1}}{\left(\prod_{r=0}^{n_{1}}{\left(1-{\left({\left(\phi \right)}_{ji}^{r}\right)}^{q}\right)}^{\left(p_{ji}^{r}\alpha_{r}\right)}\right)}^{\beta_{i}}\right)}^{\gamma_{j}}},\prod_{j=1}^{m_{1}}{\left(\prod_{i=1}^{l_{1}}{\left(\prod_{r=0}^{n_{1}}{\left({\left(\varphi \right)}_{ji}^{r}\right)}^{\left(p_{ji}^{r}\alpha_{r}\right)}\right)}^{\beta_{i}}\right)}^{\gamma_{j}}\rangle \\ ⊕\langle \sqrt[{q}]{1-{\left({\left({\left(1-{\left({\left(\phi \right)}_{\left(l_{1}+1)(n_{1}+1\right)}^{m_{1}+1}\right)}^{q}\right)}^{\left((p_{\left(m_{1}+1)(l_{1}+1\right)}^{\left(n_{1}+1\right)})(\alpha_{\left(n_{1}+1\right)})\right)}\right)}^{\beta_{\left(l_{1}+1\right)}}\right)}^{\gamma_{\left(m_{1}+1\right)}}},\\ {\left({\left({\left({\left(\varphi \right)}_{\left(l_{1}+1)(n_{1}+1\right)}^{\left(m_{1}+1\right)}\right)}^{\left((p_{\left(m_{1}+1)(l_{1}+1\right)}^{\left(n_{1}+1\right)})(\alpha_{n_{1}+1})\right)}\right)}^{\beta_{\left(l_{1}+1\right)}}\right)}^{\gamma_{\left(m_{1}+1\right)}}\rangle \end{array}\right)$

= $\langle \sqrt[{q}]{1-\prod_{j=1}^{m_{1}+1}{\left(\prod_{i=1}^{l_{1}+1}{\left(\prod_{r=0}^{n_{1}+1}{\left(1-{\left({\left(\phi \right)}_{ji}^{r}\right)}^{q}\right)}^{\left(p_{ji}^{r}\alpha_{r}\right)}\right)}^{\beta_{i}}\right)}^{\gamma_{j}}},\prod_{j=1}^{m_{1}+1}{\left(\prod_{i=1}^{l_{1}+1}{\left(\prod_{r=0}^{n_{1}+1}{\left({\left(\varphi \right)}_{ji}^{r}\right)}^{\left(p_{ji}^{r}\alpha_{r}\right)}\right)}^{\beta_{i}}\right)}^{\gamma_{j}}\rangle$

Consequently, according to the mathematical induction Theorem is true for all positive numbers. □

Example 2 Consider Example 1, where GGq-ROFNSS is expressed in Table 2. Let weighed vectors $\beta ={\left[\frac{\beta_{1}}{0.20},\frac{\beta_{2}}{0.35},\frac{\beta_{3}}{0.45}\right]}^{T}$, $\gamma ={\left[\frac{\gamma_{1}}{0.16},\frac{\gamma_{2}}{0.25},\frac{\gamma_{3}}{0.29},\frac{\gamma_{4}}{0.30}\right]}^{T}$, $\alpha ={\left[\frac{\alpha_{0}}{0.10},\frac{\alpha_{1}}{0.15},\frac{\alpha_{1}}{0.35},\frac{\alpha_{2}}{0.40}\right]}^{T}$ for B,U and set of moderators plus q-ROFNSS, respectively. Take q=4, by Theorem 3.9, we calculate the following:

$\sqrt[{q}]{1-\prod_{j=1}^{m}{\left(\prod_{i=1}^{l}{\left(\prod_{r=0}^{n}{\left(1-{\left({\left(\phi \right)}_{ji}^{r}\right)}^{q}\right)}^{\left(p_{ji}^{r}\alpha_{r}\right)}\right)}^{\beta_{i}}\right)}^{\gamma_{j}}}$

$=\sqrt[{4}]{1-\left(\begin{array}{c} {\left(\begin{array}{c} {\left({\left(1-{\left(0.10\right)}^{4}\right)}^{0.10\times 2}{\left(1-{\left(0.09\right)}^{4}\right)}^{0.15\times 3}{\left(1-{\left(0.20\right)}^{4}\right)}^{0.35\times 1}{\left(1-{\left(0.24\right)}^{4}\right)}^{0.40\times 3}\right)}^{0.20}\\ {\left({\left(1-{\left(0.25\right)}^{4}\right)}^{0.10\times 3}{\left(1-{\left(0.05\right)}^{4}\right)}^{0.15\times 4}{\left(1-{\left(0.30\right)}^{4}\right)}^{0.35\times 3}{\left(1-{\left(0.12\right)}^{4}\right)}^{0.40\times 4}\right)}^{0.35}\\ {\left({\left(1-{\left(0.31\right)}^{4}\right)}^{0.10\times 2}{\left(1-{\left(0.13\right)}^{4}\right)}^{0.15\times 2}{\left(1-{\left(0.42\right)}^{4}\right)}^{0.35\times 2}{\left(1-{\left(0.27\right)}^{4}\right)}^{0.40\times 3}\right)}^{0.45} \end{array}\right)}^{0.16}\\ {\left(\begin{array}{c} {\left({\left(1-{\left(0.13\right)}^{4}\right)}^{0.10\times 1}{\left(1-{\left(0.09\right)}^{4}\right)}^{0.15\times 3}{\left(1-{\left(0.20\right)}^{4}\right)}^{0.35\times 1}{\left(1-{\left(0.24\right)}^{4}\right)}^{0.40\times 3}\right)}^{0.20}\\ {\left({\left(1-{\left(0.35\right)}^{4}\right)}^{0.10\times 2}{\left(1-{\left(0.05\right)}^{4}\right)}^{0.15\times 4}{\left(1-{\left(0.30\right)}^{4}\right)}^{0.35\times 3}{\left(1-{\left(0.12\right)}^{4}\right)}^{0.40\times 4}\right)}^{0.35}\\ {\left({\left(1-{\left(0.30\right)}^{4}\right)}^{0.10\times 3}{\left(1-{\left(0.13\right)}^{4}\right)}^{0.15\times 2}{\left(1-{\left(0.42\right)}^{4}\right)}^{0.35\times 2}{\left(1-{\left(0.27\right)}^{4}\right)}^{0.40\times 3}\right)}^{0.45} \end{array}\right)}^{0.25}\\ {\left(\begin{array}{c} {\left({\left(1-{\left(0.23\right)}^{4}\right)}^{0.10\times 5}{\left(1-{\left(0.09\right)}^{4}\right)}^{0.15\times 3}{\left(1-{\left(0.20\right)}^{4}\right)}^{0.35\times 1}{\left(1-{\left(0.24\right)}^{4}\right)}^{0.40\times 3}\right)}^{0.20}\\ {\left({\left(1-{\left(0.26\right)}^{4}\right)}^{0.10\times 4}{\left(1-{\left(0.05\right)}^{4}\right)}^{0.15\times 4}{\left(1-{\left(0.30\right)}^{4}\right)}^{0.35\times 3}{\left(1-{\left(0.12\right)}^{4}\right)}^{0.40\times 4}\right)}^{0.35}\\ {\left({\left(1-{\left(0.29\right)}^{4}\right)}^{0.10\times 2}{\left(1-{\left(0.13\right)}^{4}\right)}^{0.15\times 2}{\left(1-{\left(0.42\right)}^{4}\right)}^{0.35\times 2}{\left(1-{\left(0.27\right)}^{4}\right)}^{0.40\times 3}\right)}^{0.45} \end{array}\right)}^{0.29}\\ {\left(\begin{array}{c} {\left({\left(1-{\left(0.21\right)}^{4}\right)}^{0.10\times 4}{\left(1-{\left(0.09\right)}^{4}\right)}^{0.15\times 3}{\left(1-{\left(0.20\right)}^{4}\right)}^{0.35\times 1}{\left(1-{\left(0.24\right)}^{4}\right)}^{0.40\times 3}\right)}^{0.20}\\ {\left({\left(1-{\left(0.36\right)}^{4}\right)}^{0.10\times 0}{\left(1-{\left(0.05\right)}^{4}\right)}^{0.15\times 4}{\left(1-{\left(0.30\right)}^{4}\right)}^{0.35\times 3}{\left(1-{\left(0.12\right)}^{4}\right)}^{0.40\times 4}\right)}^{0.35}\\ {\left({\left(1-{\left(0.19\right)}^{4}\right)}^{0.10\times 1}{\left(1-{\left(0.13\right)}^{4}\right)}^{0.15\times 2}{\left(1-{\left(0.42\right)}^{4}\right)}^{0.35\times 2}{\left(1-{\left(0.27\right)}^{4}\right)}^{0.40\times 3}\right)}^{0.45} \end{array}\right)}^{0.30} \end{array}\right)}$

$\sqrt[{q}]{1-\prod_{j=1}^{m}{\left(\prod_{i=1}^{l}{\left(\prod_{r=0}^{n}{\left(1-{\left({\left(\phi \right)}_{ji}^{r}\right)}^{q}\right)}^{\left(p_{ji}^{r}\alpha_{r}\right)}\right)}^{\beta_{i}}\right)}^{\gamma_{j}}}=0.3495$,

$\prod_{j=1}^{m}{\left(\prod_{i=1}^{l}{\left(\prod_{r=0}^{n}{\left({\left(\varphi \right)}_{ji}^{r}\right)}^{\left(p_{ji}^{r}\alpha_{r}\right)}\right)}^{\beta_{i}}\right)}^{\gamma_{j}}$

= $\left(\begin{array}{c} {\left(\begin{array}{c} {\left({\left(0.12\right)}^{0.10\times 2}{\left(0.71\right)}^{0.15\times 3}{\left(0.30\right)}^{0.35\times 1}{\left(0.28\right)}^{0.40\times 3}\right)}^{0.20}\\ {\left({\left(0.35\right)}^{0.10\times 3}{\left(0.65\right)}^{0.15\times 4}{\left(0.40\right)}^{0.35\times 3}{\left(0.16\right)}^{0.40\times 4}\right)}^{0.35}\\ {\left({\left(0.37\right)}^{0.10\times 2}{\left(0.37\right)}^{0.15\times 2}{\left(0.50\right)}^{0.35\times 2}{\left(0.44\right)}^{0.40\times 3}\right)}^{0.45} \end{array}\right)}^{0.16}{\left(\begin{array}{c} {\left({\left(0.23\right)}^{0.10\times 1}{\left(0.71\right)}^{0.15\times 3}{\left(0.30\right)}^{0.35\times 1}{\left(0.28\right)}^{0.40\times 3}\right)}^{0.20}\\ {\left({\left(0.45\right)}^{0.10\times 2}{\left(0.65\right)}^{0.15\times 4}{\left(0.40\right)}^{0.35\times 3}{\left(0.16\right)}^{0.40\times 4}\right)}^{0.35}\\ {\left({\left(0.39\right)}^{0.10\times 3}{\left(0.37\right)}^{0.15\times 2}{\left(0.50\right)}^{0.35\times 2}{\left(0.44\right)}^{0.40\times 3}\right)}^{0.45} \end{array}\right)}^{0.25}\\ {\left(\begin{array}{c} {\left({\left(0.28\right)}^{0.10\times 5}{\left(0.71\right)}^{0.15\times 3}{\left(0.30\right)}^{0.35\times 1}{\left(0.28\right)}^{0.40\times 3}\right)}^{0.20}\\ {\left({\left(0.28\right)}^{0.10\times 4}{\left(0.65\right)}^{0.15\times 4}{\left(0.40\right)}^{0.35\times 3}{\left(0.16\right)}^{0.40\times 4}\right)}^{0.35}\\ {\left({\left(0.33\right)}^{0.10\times 2}{\left(0.37\right)}^{0.15\times 2}{\left(0.50\right)}^{0.35\times 2}{\left(0.44\right)}^{0.40\times 3}\right)}^{0.45} \end{array}\right)}^{0.29}{\left(\begin{array}{c} {\left({\left(0.41\right)}^{0.10\times 4}{\left(0.71\right)}^{0.15\times 3}{\left(0.30\right)}^{0.35\times 1}{\left(0.28\right)}^{0.40\times 3}\right)}^{0.20}\\ {\left({\left(0.44\right)}^{0.10\times 0}{\left(0.65\right)}^{0.15\times 4}{\left(0.40\right)}^{0.35\times 3}{\left(0.16\right)}^{0.40\times 4}\right)}^{0.35}\\ {\left({\left(0.31\right)}^{0.10\times 1}{\left(0.37\right)}^{0.15\times 2}{\left(0.50\right)}^{0.35\times 2}{\left(0.44\right)}^{0.40\times 3}\right)}^{0.45} \end{array}\right)}^{0.30} \end{array}\right)$

$\prod_{j=1}^{m}{\left(\prod_{i=1}^{l}{\left(\prod_{r=0}^{n}{\left({\left(\varphi \right)}_{ji}^{r}\right)}^{\left(p_{ji}^{r}\alpha_{r}\right)}\right)}^{\beta_{i}}\right)}^{\gamma_{j}}=0.0537$. Hence $WA^{GGq-ROFNSS}=\langle 0.3495,0.0537\rangle$.

Theorem 3.10 *Idempotency:*

*If* ${\overset{˜}{\tau }}_{ji}^{r}=\langle {\left(\phi \right)}_{ji}^{r},{\left(\varphi \right)}_{ji}^{r}\rangle =\langle \phi ,\varphi \rangle$ *, and* $p_{b}=p_{ji}^{r}=1$ ∀(i=1,2,3,⋯,l,j=1,2,⋯,m) *and* r=0,1,⋯,n *. Then* $WA^{GGq-ROFNSS}={⊕}_{j=1}^{m}\gamma_{j}({⊕}_{i=1}^{l}\beta_{i}({⊕}_{r=0}^{n}(\alpha_{r}p_{ji}^{r}){\overset{˜}{\tau }}_{ji}^{r}))=\langle \phi ,\varphi \rangle$ *.*

Proof Given that ${\overset{˜}{\tau }}_{ji}^{r}=\langle {\left(\phi \right)}_{ji}^{r},{\left(\varphi \right)}_{ji}^{r}\rangle =\langle \phi ,\varphi \rangle$. Then $WA^{GGq-ROFNS}=$

$\left(\begin{array}{c} \langle \sqrt[{q}]{1-\prod_{j=1}^{m}{\left(\prod_{i=1}^{l}{\left(\prod_{r=0}^{n}{\left(1-{\left({\left(\phi \right)}_{ji}^{r}\right)}^{q}\right)}^{\left(p_{ji}^{r}\alpha_{r}\right)}\right)}^{\beta_{i}}\right)}^{\gamma_{j}}},\prod_{j=1}^{m}{\left(\prod_{i=1}^{l}{\left(\prod_{r=0}^{n}{\left({\left(\varphi \right)}_{ji}^{r}\right)}^{\left(p_{ji}^{r}\alpha_{r}\right)}\right)}^{\beta_{i}}\right)}^{\gamma_{j}}\rangle \end{array}\right)$

= $\left(\begin{array}{c} \langle \sqrt[{q}]{1-{\left({\left({\left(1-{\left(\phi \right)}^{q}\right)}^{\sum_{r=0}^{n}\alpha_{r}=1}\right)}^{\sum_{i=1}^{l}\beta_{i}=1}\right)}^{\sum_{j=1}^{m}\gamma_{j}=1}},\\ {\left({\left({\left(\varphi \right)}^{\sum_{r=0}^{n}\alpha_{r}=1}\right)}^{\sum_{i=1}^{l}(\beta_{i}=1)}\right)}^{\sum_{j=1}^{m}\gamma_{j}=1}\rangle \end{array}\right)=\langle \sqrt[{q}]{1-(1-{\left(\phi \right)}^{q})},\varphi \rangle =\langle \phi ,\varphi \rangle$. □

Theorem 3.11 *Monotonicity:*

*Let two GGq-ROFNSSs*${\left(\overset{˜}{\tau }\right)}_{ji}^{r}$*,*${\left({\overset{˜}{\tau }}^{\prime }\right)}_{ji}^{r}$(i=1,2,⋯,l,j=1,2,⋯,m,r=0,1,2,⋯,n)*over*U*. If*${\left(\overset{˜}{\tau }\right)}_{ji}^{r}\leq {\left({\overset{˜}{\tau }}^{\prime }\right)}_{ji}^{r}$ ∀ i=1,2,⋯,l,j=1,2,⋯,m,r=0,1,2,⋯,n *then*

$WA^{GGq-ROFNSS}({\left(\overset{˜}{\tau }\right)}_{ji}^{r})\leq WA^{GGq-ROFNSS}({\left({\overset{˜}{\tau }}^{\prime }\right)}_{ji}^{r})$ *.*

Proof Given that ${\left(\overset{˜}{\tau }\right)}_{ji}^{r}\leq {\left({\overset{˜}{\tau }}^{\prime }\right)}_{ji}^{r}$ ∀ i=1,2,⋯,l,j=1,2,⋯,m,r=0,1,2,⋯,n. Then ${\left(\phi \right)}_{ji}^{r}\leq {\left(\phi^{\prime }\right)}_{ji}^{r}$, ${\left(\varphi \right)}_{ji}^{r}\geq {\left(\varphi^{\prime }\right)}_{ji}^{r}$. So that

$1-{\left(\phi \right)}_{ji}^{r}\geq 1-{\left(\phi^{\prime }\right)}_{ji}^{r}$

⇔ $\sqrt[{q}]{1-\prod_{j=1}^{m}{\left(\prod_{i=1}^{l}{\left(\prod_{r=0}^{n}{\left(1-{\left({\left(\phi \right)}_{ji}^{r}\right)}^{q}\right)}^{\left(p_{ji}^{r}\alpha_{r}\right)}\right)}^{\beta_{i}}\right)}^{\gamma_{j}}}$

$\leq \sqrt[{q}]{1-\prod_{j=1}^{m}{\left(\prod_{i=1}^{l}{\left(\prod_{r=0}^{n}{\left(1-{\left({\left(\phi^{\prime }\right)}_{ji}^{r}\right)}^{q}\right)}^{\left(p_{ji}^{r}\alpha_{r}\right)}\right)}^{\beta_{i}}\right)}^{\gamma_{j}}}$.

Also $\prod_{j=1}^{m}{\left(\prod_{i=1}^{l}{\left(\prod_{r=0}^{n}{\left({\left(\varphi \right)}_{ji}^{r}\right)}^{\left(p_{ji}^{r}\alpha_{r}\right)}\right)}^{\beta_{i}}\right)}^{\gamma_{j}}$ $\geq \prod_{j=1}^{m}{\left(\prod_{i=1}^{l}{\left(\prod_{r=0}^{n}{\left({\left(\varphi^{\prime }\right)}_{ji}^{r}\right)}^{\left(p_{ji}^{r}\alpha_{r}\right)}\right)}^{\beta_{i}}\right)}^{\gamma_{j}}$.

If $WA^{GGq-ROFNSS}({\left(\overset{˜}{\tau }\right)}_{ji}^{r})=\tau$ and $WA^{GGq-ROFNSS}({\left({\overset{˜}{\tau }}^{\prime }\right)}_{ji}^{r})=\tau^{\prime }$ then

$S(\tau )=1-\prod_{j=1}^{m}{\left(\prod_{i=1}^{l}{\left(\prod_{r=0}^{n}{\left(1-{\left({\left(\phi \right)}_{ji}^{r}\right)}^{q}\right)}^{\left(p_{ji}^{r}\alpha_{r}\right)}\right)}^{\beta_{i}}\right)}^{\gamma_{j}}-{\left(\prod_{j=1}^{m}{\left(\prod_{i=1}^{l}{\left(\prod_{r=0}^{n}{\left({\left(\varphi \right)}_{ji}^{r}\right)}^{\left(p_{ji}^{r}\alpha_{r}\right)}\right)}^{\beta_{i}}\right)}^{\gamma_{j}}\right)}^{q}$

$\leq 1-\prod_{j=1}^{m}{\left(\prod_{i=1}^{l}{\left(\prod_{r=0}^{n}{\left(1-{\left({\left(\phi^{\prime }\right)}_{ji}^{r}\right)}^{q}\right)}^{\left(p_{ji}^{r}\alpha_{r}\right)}\right)}^{\beta_{i}}\right)}^{\gamma_{j}}-{\left(\prod_{j=1}^{m}{\left(\prod_{i=1}^{l}{\left(\prod_{r=0}^{n}{\left({\left(\varphi^{\prime }\right)}_{ji}^{r}\right)}^{\left(p_{ji}^{r}\alpha_{r}\right)}\right)}^{\beta_{i}}\right)}^{\gamma_{j}}\right)}^{q}$$=S(\tau^{\prime })$.

By the Definition of score function, $S(\tau )\leq S(\tau^{\prime })$ ⇔ $\tau \leq \tau^{\prime }$.

Hence $WA^{GGq-ROFNSS}({\left(\overset{˜}{\tau }\right)}_{ji}^{r})\leq WA^{GGq-ROFNSS}({\left({\overset{˜}{\tau }}^{\prime }\right)}_{ji}^{r})$. □

Theorem 3.12 *If* ${\left({\overset{˜}{\tau }}^{+}\right)}_{ji}^{r}=\langle {\left({\overset{˜}{\phi }}^{+}\right)}_{ji}^{r},{\left({\overset{˜}{\varphi }}^{-}\right)}_{ji}^{r}\rangle =\langle max_{j}max_{i}max_{r}\{{\left(\phi \right)}_{ji}^{r}\},min_{j}min_{i}min_{r}\{{\left(\varphi \right)}_{ji}^{r}\}\rangle$ *and*

${\left({\overset{˜}{\tau }}^{-}\right)}_{ji}^{r}=\langle {\left({\overset{˜}{\phi }}^{-}\right)}_{ji}^{r},{\left({\overset{˜}{\varphi }}^{+}\right)}_{ji}^{r}\rangle =\langle min_{j}min_{i}min_{r}\{{\left(\phi \right)}_{ji}^{r}\},max_{j}max_{i}max_{r}\{{\left(\varphi \right)}_{ji}^{r}\}\rangle$ *. Then*

${\left({\overset{˜}{\tau }}^{-}\right)}_{ji}^{r}\leq WA^{GGq-ROFNSS}\leq {\left({\overset{˜}{\tau }}^{+}\right)}_{ji}^{r}$ *,* ∀(i=1,2,3,⋯,l,j=1,2,⋯,m) *and* r=0,1,2,⋯,n *.*

Proof Straightforward to the Theorem 3.10. □

**Group-based generalized q-rung orthopair fuzzy N-soft weighted geometric (GGq-ROFNSWG) operators**

The new GGq-ROFNSWG operators based on the GGq-ROFNSS illustration in Table 1, are defined in the following notion.

Definition 3.13 Let $T_{K}=(F_{q},B,N,K)$ be a GGq-ROFNSS over U as shown in Table 1. Let ${\left[\beta_{1},\beta_{2},\cdots ,\beta_{l}\right]}^{T}$ be a weighted vector over set of parameters such that $\beta_{i}>0$ and $\sum_{i=1}^{l}\beta_{i}=1$. Let weighted vector ${\left[\alpha_{0},\alpha_{1},\alpha_{2},\cdots ,\alpha_{N}\right]}^{T}$, such that $\alpha_{r}>0$ and $\sum_{r=0}^{n}\alpha_{r}=1$, where $\alpha_{1},\alpha_{2},\cdots ,\alpha_{n}$ are the weights over moderators, and $\alpha_{0}$ is the weight for the entire data set in Eq-ROFNSS (which is shown in Table 1). Let $\left[\gamma_{1},\gamma_{2},\cdots ,\gamma_{m}\right]$ be the set of essential weights of alternatives with condition $\gamma_{m}>0$ and $\sum_{j=1}^{m}\gamma_{m}=1$. Let r=0,1,2,⋯,n, then the GGq-ROFNSWG operators’ manifested as follows. $\Lambda =WG^{GGq-ROFNSS}={⊗}_{j=1}^{m}\gamma_{j}({⊗}_{i=1}^{l}\beta_{i}({⊗}_{r=0}^{n}(\alpha_{r}p_{ji}^{r}).{\overset{˜}{\tau }}_{ji}^{r}))$ where $WG^{GGq-ROFNSS}$ denoted GGq-ROFNSGA operators on GGq-ROFNSS.

Theorem 3.14 *Suppose that* ${\overset{˜}{\tau }}_{ij}^{r}=\langle {\left(\varphi \right)}_{ji}^{r},{\left(\phi \right)}_{ji}^{r}\rangle$ *, be a GGq-ROFNSVs. So* (i=1,2,3,⋯,l,j=0,1,2,⋯,m) *and* r=1,2,3,⋯,n *. where GGq-ROFNSGA operator is given below as;*

$WG^{GGq-ROFNSS}={⊗}_{j=1}^{m}\gamma_{j}({⊗}_{i=1}^{l}\beta_{i}({⊗}_{r=0}^{n}(\alpha_{r}p_{ji}^{r}){\overset{˜}{\tau }}_{ji}^{r}))=$

$\left(\begin{array}{c} \langle \prod_{j=1}^{m}{\left(\prod_{i=1}^{l}{\left(\prod_{r=1}^{n}{\left({\left(\phi \right)}_{ji}^{r}\right)}^{\left(p_{ji}^{r}\alpha_{r}\right)}\right)}^{\beta_{i}}\right)}^{\gamma_{j}},\sqrt[{q}]{1-\prod_{j=1}^{m}{\left(\prod_{i=1}^{l}{\left(\prod_{r=0}^{n}{\left(1-{\left({\left(\varphi \right)}_{ji}^{r}\right)}^{q}\right)}^{\left(p_{ji}^{r}\alpha_{r}\right)}\right)}^{\beta_{i}}\right)}^{\gamma_{j}}}\rangle \end{array}\right)$ *.*

Proof It is comparable to the Theorem 3.9. □

Example 3 Consider Example 1, where GGq-ROFNSS is expressed in Table 2. Take q=4, by Theorem 3.14, we calculate the following

$\prod_{j=1}^{m}{\left(\prod_{i=1}^{l}{\left(\prod_{r=1}^{n}{\left({\left(\phi \right)}_{ji}^{r}\right)}^{\left(p_{ji}^{r}\alpha_{r}\right)}\right)}^{\beta_{i}}\right)}^{\gamma_{j}}$

= $\left(\begin{array}{c} {\left(\begin{array}{c} {\left({\left(0.10\right)}^{0.10\times 2}{\left(0.09\right)}^{0.15\times 3}{\left(0.20\right)}^{0.35\times 1}{\left(0.24\right)}^{0.40\times 3}\right)}^{0.20}\\ {\left({\left(0.25\right)}^{0.10\times 3}{\left(0.05\right)}^{0.15\times 4}{\left(0.30\right)}^{0.35\times 3}{\left(0.12\right)}^{0.40\times 4}\right)}^{0.35}\\ {\left({\left(0.31\right)}^{0.10\times 2}{\left(0.13\right)}^{0.15\times 2}{\left(0.42\right)}^{0.35\times 2}{\left(0.27\right)}^{0.40\times 3}\right)}^{0.45} \end{array}\right)}^{0.16}{\left(\begin{array}{c} {\left({\left(0.13\right)}^{0.10\times 1}{\left(0.09\right)}^{0.15\times 3}{\left(0.20\right)}^{0.35\times 1}{\left(0.24\right)}^{0.40\times 3}\right)}^{0.20}\\ {\left({\left(0.35\right)}^{0.10\times 2}{\left(0.05\right)}^{0.15\times 4}{\left(0.30\right)}^{0.35\times 3}{\left(0.12\right)}^{0.40\times 4}\right)}^{0.35}\\ {\left({\left(0.30\right)}^{0.10\times 3}{\left(0.13\right)}^{0.15\times 2}{\left(0.42\right)}^{0.35\times 2}{\left(0.27\right)}^{0.40\times 3}\right)}^{0.45} \end{array}\right)}^{0.25}\\ {\left(\begin{array}{c} {\left({\left(0.23\right)}^{0.10\times 5}{\left(0.09\right)}^{0.15\times 3}{\left(0.20\right)}^{0.35\times 1}{\left(0.24\right)}^{0.40\times 3}\right)}^{0.20}\\ {\left({\left(0.26\right)}^{0.10\times 4}{\left(0.05\right)}^{0.15\times 4}{\left(0.30\right)}^{0.35\times 3}{\left(0.12\right)}^{0.40\times 4}\right)}^{0.35}\\ {\left({\left(0.29\right)}^{0.10\times 2}{\left(0.13\right)}^{0.15\times 2}{\left(0.42\right)}^{0.35\times 2}{\left(0.27\right)}^{0.40\times 3}\right)}^{0.45} \end{array}\right)}^{0.29}{\left(\begin{array}{c} {\left({\left(0.21\right)}^{0.10\times 4}{\left(0.09\right)}^{0.15\times 3}{\left(0.20\right)}^{0.35\times 1}{\left(0.24\right)}^{0.40\times 3}\right)}^{0.20}\\ {\left({\left(0.36\right)}^{0.10\times 0}{\left(0.05\right)}^{0.15\times 4}{\left(0.30\right)}^{0.35\times 3}{\left(0.12\right)}^{0.40\times 4}\right)}^{0.35}\\ {\left({\left(0.19\right)}^{0.10\times 1}{\left(0.13\right)}^{0.15\times 2}{\left(0.42\right)}^{0.35\times 2}{\left(0.27\right)}^{0.40\times 3}\right)}^{0.45} \end{array}\right)}^{0.30} \end{array}\right)$

$\prod_{j=1}^{m}{\left(\prod_{i=1}^{l}{\left(\prod_{r=1}^{n}{\left({\left(\phi \right)}_{ji}^{r}\right)}^{\left(p_{ji}^{r}\alpha_{r}\right)}\right)}^{\beta_{i}}\right)}^{\gamma_{j}}=0.0111$, $\sqrt[{q}]{1-\prod_{j=1}^{m}{\left(\prod_{i=1}^{l}{\left(\prod_{r=0}^{n}{\left(1-{\left({\left(\varphi \right)}_{ji}^{r}\right)}^{q}\right)}^{\left(p_{ji}^{r}\alpha_{r}\right)}\right)}^{\beta_{i}}\right)}^{\gamma_{j}}}$

$=\sqrt[{4}]{1-\left(\begin{array}{c} {\left(\begin{array}{c} {\left({\left(1-{\left(0.12\right)}^{4}\right)}^{0.10\times 2}{\left(1-{\left(0.71\right)}^{4}\right)}^{0.15\times 3}{\left(1-{\left(0.30\right)}^{4}\right)}^{0.35\times 1}{\left(1-{\left(0.28\right)}^{4}\right)}^{0.40\times 3}\right)}^{0.20}\\ {\left({\left(1-{\left(0.35\right)}^{4}\right)}^{0.10\times 3}{\left(1-{\left(0.65\right)}^{4}\right)}^{0.15\times 4}{\left(1-{\left(0.40\right)}^{4}\right)}^{0.35\times 3}{\left(1-{\left(0.16\right)}^{4}\right)}^{0.40\times 4}\right)}^{0.35}\\ {\left({\left(1-{\left(0.37\right)}^{4}\right)}^{0.10\times 2}{\left(1-{\left(0.37\right)}^{4}\right)}^{0.15\times 2}{\left(1-{\left(0.50\right)}^{4}\right)}^{0.35\times 2}{\left(1-{\left(0.44\right)}^{4}\right)}^{0.40\times 3}\right)}^{0.45} \end{array}\right)}^{0.16}\\ {\left(\begin{array}{c} {\left({\left(1-{\left(0.23\right)}^{4}\right)}^{0.10\times 1}{\left(1-{\left(0.71\right)}^{4}\right)}^{0.15\times 3}{\left(1-{\left(0.30\right)}^{4}\right)}^{0.35\times 1}{\left(1-{\left(0.28\right)}^{4}\right)}^{0.40\times 3}\right)}^{0.20}\\ {\left({\left(1-{\left(0.45\right)}^{4}\right)}^{0.10\times 2}{\left(1-{\left(0.65\right)}^{4}\right)}^{0.15\times 4}{\left(1-{\left(0.40\right)}^{4}\right)}^{0.35\times 3}{\left(1-{\left(0.16\right)}^{4}\right)}^{0.40\times 4}\right)}^{0.35}\\ {\left({\left(1-{\left(0.39\right)}^{4}\right)}^{0.10\times 3}{\left(1-{\left(0.37\right)}^{4}\right)}^{0.15\times 2}{\left(1-{\left(0.50\right)}^{4}\right)}^{0.35\times 2}{\left(1-{\left(0.44\right)}^{4}\right)}^{0.40\times 3}\right)}^{0.45} \end{array}\right)}^{0.25}\\ {\left(\begin{array}{c} {\left({\left(1-{\left(0.28\right)}^{4}\right)}^{0.10\times 5}{\left(1-{\left(0.71\right)}^{4}\right)}^{0.15\times 3}{\left(1-{\left(0.30\right)}^{4}\right)}^{0.35\times 1}{\left(1-{\left(0.28\right)}^{4}\right)}^{0.40\times 3}\right)}^{0.20}\\ {\left({\left(1-{\left(0.28\right)}^{4}\right)}^{0.10\times 4}{\left(1-{\left(0.65\right)}^{4}\right)}^{0.15\times 4}{\left(1-{\left(0.40\right)}^{4}\right)}^{0.35\times 3}{\left(1-{\left(0.16\right)}^{4}\right)}^{0.40\times 4}\right)}^{0.35}\\ {\left({\left(1-{\left(0.33\right)}^{4}\right)}^{0.10\times 2}{\left(1-{\left(0.37\right)}^{4}\right)}^{0.15\times 2}{\left(1-{\left(0.50\right)}^{4}\right)}^{0.35\times 2}{\left(1-{\left(0.44\right)}^{4}\right)}^{0.40\times 3}\right)}^{0.45} \end{array}\right)}^{0.29}\\ {\left(\begin{array}{c} {\left({\left(1-{\left(0.41\right)}^{4}\right)}^{0.10\times 4}{\left(1-{\left(0.71\right)}^{4}\right)}^{0.15\times 3}{\left(1-{\left(0.30\right)}^{4}\right)}^{0.35\times 1}{\left(1-{\left(0.28\right)}^{4}\right)}^{0.40\times 3}\right)}^{0.20}\\ {\left({\left(1-{\left(0.44\right)}^{4}\right)}^{0.10\times 0}{\left(1-{\left(0.65\right)}^{4}\right)}^{0.15\times 4}{\left(1-{\left(0.40\right)}^{4}\right)}^{0.35\times 3}{\left(1-{\left(0.16\right)}^{4}\right)}^{0.40\times 4}\right)}^{0.35}\\ {\left({\left(1-{\left(0.31\right)}^{4}\right)}^{0.10\times 1}{\left(1-{\left(0.37\right)}^{4}\right)}^{0.15\times 2}{\left(1-{\left(0.50\right)}^{4}\right)}^{0.35\times 2}{\left(1-{\left(0.44\right)}^{4}\right)}^{0.40\times 3}\right)}^{0.45} \end{array}\right)}^{0.30} \end{array}\right)}$

$\sqrt[{q}]{1-\prod_{j=1}^{m}{\left(\prod_{i=1}^{l}{\left(\prod_{r=0}^{n}{\left(1-{\left({\left(\varphi \right)}_{ji}^{r}\right)}^{q}\right)}^{\left(p_{ji}^{r}\alpha_{r}\right)}\right)}^{\beta_{i}}\right)}^{\gamma_{j}}}=0.5864$.

Hence $WG^{GGq-ROFNSS}=\langle 0.0111,0.5864\rangle$.

Theorem 3.15 *Idempotency:*

*If* ${\overset{˜}{\tau }}_{ji}^{r}=\langle {\left(\phi \right)}_{ji}^{r},{\left(\varphi \right)}_{ji}^{r}\rangle =\langle \phi ,\varphi \rangle$ *, and* $p_{b}=p_{ji}^{r}=1$ ∀(i=1,2,3,⋯,l,j=1,2,⋯,m) *and* r=0,1,⋯,n *. Then* $WG^{GGq-ROFNSS}={⊕}_{j=1}^{m}\gamma_{j}({⊕}_{i=1}^{l}\beta_{i}({⊕}_{r=0}^{n}(\alpha_{r}p_{ji}^{r}){\overset{˜}{\tau }}_{ji}^{r}))=\langle \phi ,\varphi \rangle$ *.*

Proof It is comparable to the Theorem 3.10. □

Theorem 3.16 *Boundedness:*

*If* ${\left({\overset{˜}{\tau }}^{+}\right)}_{ji}^{r}=\langle {\left({\overset{˜}{\phi }}^{+}\right)}_{ji}^{r},{\left({\overset{˜}{\varphi }}^{-}\right)}_{ji}^{r}\rangle =\langle max_{j}max_{i}max_{r}\{{\left(\phi \right)}_{ji}^{r}\},min_{j}min_{i}min_{r}\{{\left(\varphi \right)}_{ji}^{r}\}\rangle$ *and*

${\left({\overset{˜}{\tau }}^{-}\right)}_{ji}^{r}=\langle {\left({\overset{˜}{\phi }}^{-}\right)}_{ji}^{r},{\left({\overset{˜}{\varphi }}^{+}\right)}_{ji}^{r}\rangle =\langle min_{j}min_{i}min_{r}\{{\left(\phi \right)}_{ji}^{r}\},max_{j}max_{i}max_{r}\{{\left(\varphi \right)}_{ji}^{r}\}\rangle$ *. Then*

${\left({\overset{˜}{\tau }}^{-}\right)}_{ji}^{r}\leq WG^{GGq-ROFNSS}\leq {\left({\overset{˜}{\tau }}^{+}\right)}_{ji}^{r}$ *,* ∀(i=1,2,3,⋯,l,j=1,2,⋯,m) *and* r=0,1,2,⋯,n *.*

Proof It is comparable to Theorem 3.10. □

## 4 Multi-criteria decision making method

A suitable MCDM approach can be vital to manage uncertainties in each component in difficult real-world circumstances when it is difficult to make a choice based on specific criteria. A good methodology is necessary for the MCDM method on GGq-ROFNSSs since the GGq-ROFNSS combines multiple components of complicated decision-making. Therefore, a MCDM method for GGq-ROFNSSs is given as in the following;

### 4.1 MCDM procedure

In order to define a precise MCDM technique, we discussed some fundamental points as follows;

(1) Let $H=\{H_{1},H_{2},\cdots ,H_{l^{\prime }}\}$ be a set of several alternatives, each of which is related to a set of sub-alternatives $SA=\{u_{1},u_{2},\cdots ,u_{m}\}$. A set of criteria related to sub-alternatives is $B=\{c_{1},c_{2},\cdots ,c_{l}\}$.
(2) A team of experts provide assessments over B for each object in sub-alternatives $u_{j}(j=1,2.\cdots ,m)$ and given in the form of GGq-ROFNSSs
  $T_{1_{\left(K_{1}\right)}}=(F_{q}^{1},B,N,K_{1}),T_{2_{\left(K_{2}\right)}}=(F_{q}^{2},B,N,K_{2}),\cdots ,T_{{l^{\prime }}_{\left(K_{l^{\prime }}\right)}}=(F_{q}^{l^{\prime }},B,N,K_{l^{\prime }})$. Denote each GGq-ROFNSS in tables.
(3) The normalize the data
  $T_{1_{\left(K_{1}\right)}}=(F_{q}^{1},B,N,K_{1}),T_{2_{\left(K_{2}\right)}}=(F_{q}^{2},B,N,K_{2}),\cdots ,T_{{l^{\prime }}_{\left(K_{l^{\prime }}\right)}}=(F_{q}^{l^{\prime }},B,N,K_{l^{\prime }})$ in terms of parameters as follows;
  $${\overset{˜}{\tau }}_{ji}^{r}=\left\{ \begin{array}{c} ((u,p_{ji}^{r}),\;\;\langle \phi_{ji}^{r},\varphi_{ji}^{r}\rangle ),\;for\;cost\;type\;parameter,\\ ((u,p_{ji}^{r}),\;\;\langle \varphi_{ji}^{r},\phi_{ji}^{r}\rangle ),\;for\;profit\;type\;parameter, \end{array}\right.$$
  where ${\overset{˜}{\tau }}_{ji}^{r}$ represent normalized GGq-ROFNS matrices for each r=0,1,2,⋯,n.
(4) Consider the weighed vectors $\left[\beta_{1},\beta_{2},\cdots ,\beta_{l}\right]$ on B, and $\gamma ={\left[\gamma_{1},\gamma_{2},\cdots ,\gamma_{m}\right]}^{T}$ on SA. Also consider weighted vectors $\alpha ={\left[\alpha_{0},\alpha_{1},\cdots ,\alpha_{n}\right]}^{T}$, where $\alpha_{1},\alpha_{2},\cdots ,\alpha_{n}$ are the weights over moderators and $\alpha_{0}$ is the weight of the overall data in Eq-ROFNSS in each table of GGq-ROFNSSs.
(5) By applying the proposed aggregation operators (GGq-ROFNSWA by Definition 3.8 or GGq-ROFNSWG by Definition 3.13) and then obtained final GGq-ROFNSVs from each table of GGq-ROFNSSs.
(6) Calculate the score function of each GGq-ROFNSVs using the Definition 2.2.
(7) The best optimal result can be obtained on larger value of score function.

**Figure 1:**
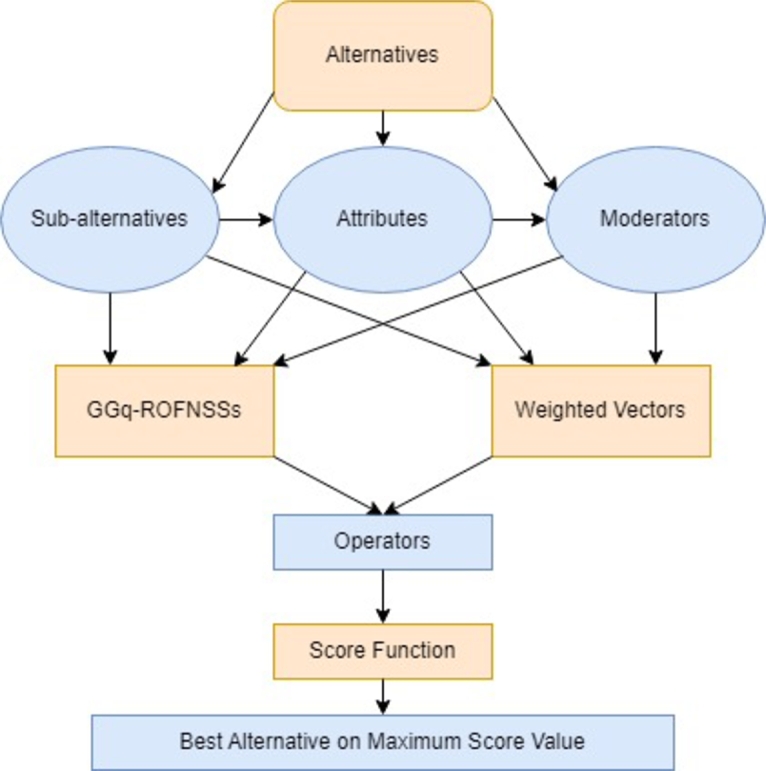
MCDM on GGq-ROFNSSs.

The projected MCDM framework is elaborated in Fig. 1.

### 4.2 Application in solar panel selection

The evaluation of solar panels for a city is a challenging process since it depends on the environment, the location of the area, the sector of needs, etc. In this study, we intended to solve a problem to evaluate a solar panel for a city. This complex problem is perfectly characterized with in the model of GGq-ROFNSSs.

A government company aims to install adequate solar panels throughout in a city in ($u_{1}$); industrial region, ($u_{2}$) rural areas, ($u_{3}$) urban areas, and ($u_{4}$) suburban areas to satisfy the city's future energy needs. The company has to choose a suitable solar panel from the set of four solar panels, $H=\{H_{1},H_{2},H_{3},H_{4}\}$. Under adverse weather conditions, solar panels are put to the test. This certifies that adverse weather, such as hail, snow, storms, and hurricanes, has little to no effect on the functioning of the solar panel. On the other hand, the company is curious about power efficiency solar power system, so it must be tested in the aforementioned locations. The company examines the impacts of temperature, hail, and hurricanes on each solar panel in each area, as well as the power efficiency of each solar panel in each area to test solar panels. For each solar panel, there are distinct elements or attributes to examine in the aforementioned four areas. As a result, the company takes into consideration four factors or parameters, namely ($c_{1}$);temperature, ($c_{2}$); Hail rating, ($c_{3}$); Hurricane rating, and ($c_{4}$); Power Efficiency, which might affect the efficiency and dependability of solar panels. Using the parameters for the aforementioned four areas, a group of specialists gives values for each solar panel in the form of q-ROFNSSs, which are shown in the upper parts of Table 6, Table 7, Table 8, Table 9, respectively. Three senior experts or moderators ($M_{1},M_{2},M_{3}$) provide values in the form of N-Pq-ROFSs for the aforementioned parameters for each solar panel. For each solar panel, these N-Pq-ROFSs are displayed in the lower parts of Table 6, Table 7, Table 8, Table 9, where $M_{1}$ checks parameter‘s effect in summer, $M_{2}$ checks parameter‘s effect in winter, and $M_{3}$ checks parameter‘s effect in autumn and spring seasons. The four Table 6, Table 7, Table 8, Table 9 are the assessment for solar panels $H_{k^{\prime }}(k^{\prime }=1,2,\cdots ,4)$, respectively. In other words we represent four GGq-ROFNSSs $T_{1_{\left(K_{1}\right)}}=(F_{q_{1}},B,N,K_{1})$,

**Table 6:** Company $H_{1}$.

U|B	*c*_1_	*c*_2_	*c*_3_	*c*_4_
*u*_1_	(1,〈0.22,0.30〉)	(4,〈0.35,0.38〉)	(3,〈0.23,0.32〉)	(5,〈0.46,0.47〉)
*u*_2_	(4,〈0.13,0.16〉)	(0,〈0.35,0.41〉)	(4,〈0.30,0.33〉)	(3,〈0.31,0.34〉)
*u*_3_	(2,〈0.23,0.25〉)	(3,〈0.26,0.27〉)	(2,〈0.29,0.35〉)	(1,〈0.32,0.36〉)
*u*_4_	(4,〈0.21,0.26〉)	(1,〈0.36,0.39〉)	(2,〈0.19,0.21〉)	(5,〈0.33,0.37〉)

$K_{M_{1}}$	(5,〈0.19,0.23〉)	(3,〈0.25,0.35〉)	(2,〈0.13,0.17〉)	(5,〈0.31,0.34〉)
$K_{M_{2}}$	(3,〈0.21,0.24〉)	(1,〈0.19,0.28〉)	(1,〈0.32,0.42〉)	(4,〈0.21,0.29〉)
$K_{M_{3}}$	(2,〈0.34,0.40〉)	(0,〈0.11,0.16〉)	(3,〈0.10,0.14〉)	(1,〈0.12,0.18〉)

**Table 7:** Company $H_{2}$.

U|B	*c*_1_	*c*_2_	*c*_3_	*c*_4_
*u*_1_	(4,〈0.13,0.34〉)	(3,〈0.35,0.52〉)	(2,〈0.32,0.56〉)	(3,〈0.44,0.47〉)
*u*_2_	(1,〈0.12,0.43〉)	(2,〈0.35,0.64〉)	(3,〈0.17,0.49〉)	(0,〈0.31,0.57〉)
*u*_3_	(5,〈0.21,0.38〉)	(4,〈0.27,0.28〉)	(2,〈0.29,0.33〉)	(1,〈0.32,0.39〉)
*u*_4_	(4,〈0.21,0.61〉)	(2,〈0.34,0.63〉)	(1,〈0.39,0.51〉)	(3,〈0.41,0.47〉)

$K_{M_{1}}$	(1,〈0.19,0.71〉)	(4,〈0.15,0.75〉)	(3,〈0.23,0.31〉)	(2,〈0.22,0.35〉)
$K_{M_{2}}$	(3,〈0.20,0.30〉)	(3,〈0.32,0.42〉)	(2,〈0.42,0.50〉)	(0,〈0.11,0.17〉)
$K_{M_{3}}$	(5,〈0.14,0.29〉)	(1,〈0.32,0.46〉)	(5,〈0.20,0.24〉)	(3,〈0.21,0.32〉)

**Table 8:** Company $H_{3}$.

U|B	*c*_1_	*c*_2_	*c*_3_	*c*_4_
*u*_1_	(4,〈0.14,0.32〉)	(3,〈0.24,0.53〉)	(4,〈0.36,0.47〉)	(0,〈0.35,0.41〉)
*u*_2_	(1,〈0.33,0.43〉)	(2,〈0.32,0.47〉)	(2,〈0.22,0.34〉)	(4,〈0.26,0.57〉)
*u*_3_	(5,〈0.43,0.56〉)	(4,〈0.25,0.48〉)	(5,〈0.28,0.63〉)	(1,〈0.30,0.39〉)
*u*_4_	(2,〈0.41,0.51〉)	(0,〈0.12,0.84〉)	(3,〈0.20,0.41〉)	(5,〈0.32,0.47〉)

$K_{M_{1}}$	(3,〈0.16,0.71〉)	(4,〈0.25,0.65〉)	(0,〈0.23,0.37〉)	(5,〈0.35,0.47〉)
$K_{M_{2}}$	(4,〈0.24,0.35〉)	(3,〈0.36,0.42〉)	(1,〈0.32,0.40〉)	(2,〈0.41,0.57〉)
$K_{M_{3}}$	(1,〈0.32,0.38〉)	(5,〈0.14,0.18〉)	(2,〈0.26,0.64〉)	(4,〈0.27,0.56〉)

**Table 9:** Company $H_{4}$.

U|B	*c*_1_	*c*_2_	*c*_3_	*c*_4_
*u*_1_	(1,〈0.31,0.39〉)	(2,〈0.35,0.45〉)	(3,〈0.43,0.53〉)	(4,〈0.22,0.37〉)
*u*_2_	(5,〈0.45,0.49〉)	(4,〈0.34,0.56〉)	(2,〈0.28,0.54〉)	(2,〈0.21,0.60〉)
*u*_3_	(3,〈0.27,0.36〉)	(3,〈0.44,0.51〉)	(4,〈0.23,0.34〉)	(1,〈0.13,0.32〉)
*u*_4_	(4,〈0.06,0.64〉)	(0,〈0.04,0.44〉)	(1,〈0.20,0.26〉)	(3,〈0.12,0.65〉)

$K_{M_{1}}$	(3,〈0.23,0.27〉)	(4,〈0.15,0.45〉)	(2,〈0.45,0.49〉)	(2,〈0.39,0.59〉)
$K_{M_{2}}$	(1,〈0.20,0.32〉)	(3,〈0.40,0.41〉)	(4,〈0.33,0.47〉)	(3,〈0.36,0.44〉)
$K_{M_{3}}$	(4,〈0.34,0.38〉)	(1,〈0.42,0.48〉)	(3,〈0.14,0.24〉)	(5,〈0.27,0.29〉)

$T_{2_{\left(K_{2}\right)}}=(F_{q_{2}},B,N,K_{2})$, $T_{3_{\left(K_{3}\right)}}=(F_{q_{3}},B,N,K_{3})$, $T_{4_{\left(K_{4}\right)}}=(F_{q_{4}},B,N,K_{4})$ in Table 6, Table 7, Table 8, Table 9 respectively. Consider the weighed vectors $\left[\frac{\gamma_{1}}{0.15},\frac{\gamma_{2}}{0.22},\frac{\gamma_{3}}{0.25},\frac{\gamma_{4}}{0.35}\right]$ for the set of areas $U=\{u_{1},u_{2},u_{3},u_{4}\}$ and $\left[\frac{\beta_{1}}{0.17},\frac{\beta_{2}}{0.28},\frac{\beta_{3}}{0.25},\frac{\beta_{4}}{0.30}\right]$ for the set of parameters $B=\{c_{1},c_{2},c_{3},c_{4}\}$. Also, consider a weighted vector $\left[\frac{\alpha_{0}}{0.18},\frac{\alpha_{1}}{0.24},\frac{\alpha_{2}}{0.26},\frac{\alpha_{3}}{0.32}\right]$ on the q-ROFNSS and N-Pq-ROFNSs of the senior experts. In other words $\alpha_{0}$ is the weight on overall data in upper half and $\frac{\alpha_{1}}{0.24},\frac{\alpha_{2}}{0.26},\frac{\alpha_{3}}{0.32}$ are the weights on senior experts $M_{r^{\prime }}(1,2,3)$, respectively. We take N=1,2,⋯,5 and q=5. Therefore the group based generalized q-rung orthopair fuzzy 6-soft sets decision matrices are shown in Table 6, Table 7, Table 8, Table 9.

### 4.3 Steps of decision making

(1) We normalized the data in Table 6, Table 7, Table 8, Table 9. As each criterion is a beneficial type parameter, thus, normalization gives same data as in Table 6, Table 7, Table 8, Table 9.
(2) We compute the GGq-ROFNSWA operators for Table 6, Table 7, Table 8, Table 9 by assuming q=5. Then we obtained GGq-ROFNVs for solar panels $H_{i}(i=1,2.\cdots ,4)$; $WA^{H_{1}}=\langle 0.6365,0.0474\rangle$, $WA^{H_{2}}=\langle 0.9810,0.0787\rangle$, $WA^{H_{3}}=\langle 0.6510,0.0880\rangle$, $WA^{H_{4}}=\langle 0.5657,0.0665\rangle$.
(3) We get score values $S(WA^{H_{1}})=0.1045$, $S(WA^{H_{2}})=0.9085$, $S(WA^{H_{3}})=0.1169$, $S(WA^{H_{4}})=0.0579$, respectively by using Definition 2.2.
(4) Then we arranged the solar panels based on aforementioned score values, that is, $H_{2}>H_{3}>H_{1}>H_{4}$.
(5) The best solar panel to implement in the city is $H_{2}$.

### 4.4 Comparative analysis

In order to compare proposed method, with existing methods we consider following examples.

**Figure 2:**
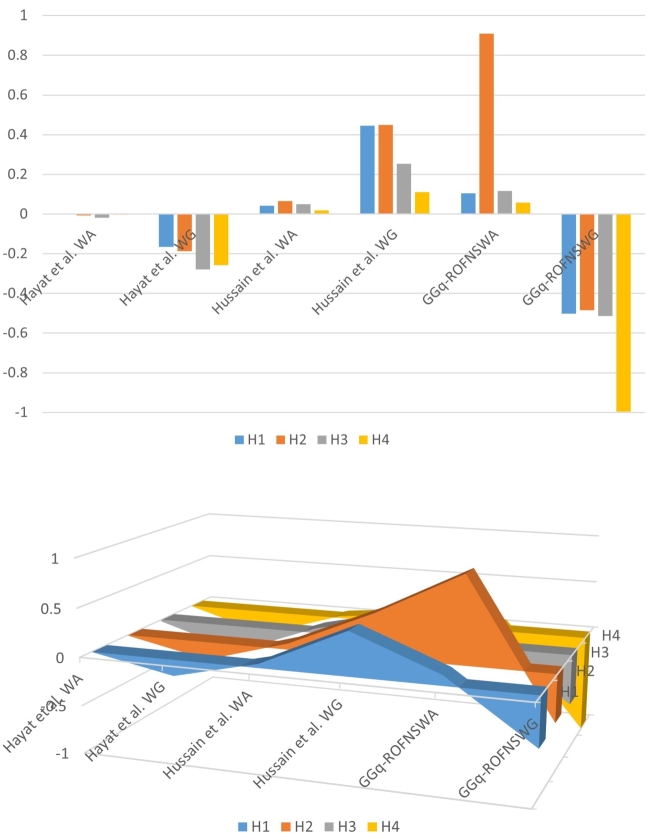
Comparison.

Example 4.1 Consider Example presented in the Section 4.2, ranking of solar panels on GGq-ROFNSWA is given by $H_{2}>H_{3}>H_{1}>H_{4}$. Now by using GGq-ROFNSWG, we obtained score values $S(WA^{H_{1}})=-0.5027$, $S(WA^{H_{2}})=-0.4842$, $S(WA^{H_{3}})=-0.5142$, $S(WA^{H_{4}})=-0.9950$, respectively by using Definition 2.2. The rankings of solar panels are then determined as $H_{2}>H_{1}>H_{3}>H_{4}$. In Table 13, comparative results between the suggested strategy and other methods are shown. Additionally, Fig. 2 offers a more logical explanation of the information in Table 13’s data.

Hayat et al. [46]'s findings are based on GGIVq-ROFSSs, however if we take one membership and one non-membership rather than an interval, this yields GGq-ROFSSs results. Thus, we compare the aforementioned method of GGq-ROF4SSs with GGq-ROFSSs by ignoring rating values in Table 6, Table 7, Table 8, Table 9 and just taking into account membership and non-membership. The rankings from this comparison analysis are shown in the upper portion of Table 13. The ranking has been somewhat altered in this instance, which is indicative of a lack of rating values. As a result, the suggested approach works better for non-binary systems with q-ROFVs.

Next, we applied the Hussian et al. [22] approach for analysis, taking into account q-ROFSSs from the upper regions of Table 6, Table 7, Table 8, Table 9. Therefore, we compare the aforementioned method of GGq-ROF4SSs with q-ROFSSs by ignoring rating values and moderators inputs in Table 6, Table 7, Table 8, Table 9 and just taking into account membership and non-membership from upper parts of Table 6, Table 7, Table 8, Table 9. The rankings from this comparison analysis are shown in the upper portion of Table 13. The ranking has been somewhat altered in this instance, which is indicative of a lack of rating values and moderators inputs in GGq-ROF4SSs. The suggested method therefore performs better for non-binary systems with q-ROFVs and moderator inputs. The suggested example in the Section 4.2 also demonstrates why it's crucial to consider moderators' advice when developing GGq-ROF4SSs.

Example 4.2 Let $H_{1}$, $H_{2}$, and $H_{3}$ be three different alternatives with a set of sub-alternatives $SA=\{u_{1},u_{2},u_{3}\}$. A set of criteria related to sub-alternatives is $B=\{c_{1},c_{2},c_{3}\}$. A group of specialists gives values in the form of q-ROFNSSs in B and SA, which are shown in the upper parts of Table 10, Table 11, Table 12. The two senior experts or moderators ($M_{1},M_{2}$) provide values in the form of N-Pq-ROFSs for the aforementioned parameters for each alternative, shown in lower parts of Table 10, Table 11, Table 12. We take N=4 and q=4. Hence three GGq-ROF4SSs are shown in Table 10, Table 11, Table 12. Consider the weighed vectors $\left[\frac{\gamma_{1}}{0.45},\frac{\gamma_{2}}{0.30},\frac{\gamma_{3}}{0.25}\right]$ for the set of sub-alternatives SA and $\left[\frac{\beta_{1}}{0.25},\frac{\beta_{2}}{0.40},\frac{\beta_{3}}{0.35}\right]$ for the set of parameters B. Also, consider a weighted vector $\left[\frac{\alpha_{0}}{0.35},\frac{\alpha_{1}}{0.33},\frac{\alpha_{2}}{0.32}\right]$ on the q-ROFNSS and N-Pq-ROFNSs of the senior experts. In other words $\frac{\alpha_{0}}{0.35}$ is the weight on overall data in upper half and $\frac{\alpha_{1}}{0.33},\frac{\alpha_{2}}{0.32}$ are the weights on senior experts $M_{r^{\prime }}(1,2)$, respectively.

**Table 10:** Alternative $H_{1}$.

U|B	*c*_1_	*c*_2_	*c*_3_
*u*_1_	(0,〈0.18,0.93〉)	(2,〈0.60,0.60〉)	(2,〈0.60,0.60〉)
*u*_2_	(3,〈0.85,0.44〉)	(1,〈0.50,0.75〉)	(3,〈0.80,0.40〉)
*u*_3_	(1,〈0.20,0.80〉)	(1,〈0.22,0.79〉)	(2,〈0.60,0.56〉)

$K_{M_{1}}$	(2,〈0.60,0.70〉)	(3,〈0.85,0.30〉)	(1,〈0.20,0.80〉)
$K_{M_{2}}$	(3,〈0.80,0.47〉)	(2,〈0.60,0.60〉)	(1,〈0.25,0.85〉)

**Table 11:** Alternative $H_{2}$.

U|B	*c*_1_	*c*_2_	*c*_3_
*u*_1_	(1,〈0.30,0.80〉)	(2,〈0.62,0.64〉)	(3,〈0.81,0.35〉)
*u*_2_	(2,〈0.79,0.70〉)	(0,〈0.16,0.92〉)	(1,〈0.26,0.72〉)
*u*_3_	(1,〈0.27,0.85〉)	(3,〈0.80,0.40〉)	(2,〈0.63,0.55〉)

$K_{M_{1}}$	(3,〈0.85,0.35〉)	(2,〈0.70,0.68〉)	(1,〈0.30,0.73〉)
$K_{M_{2}}$	(2,〈0.70,0.60〉)	(1,〈0.50,0.80〉)	(3,〈0.80,0.40〉)

**Table 12:** Alternative $H_{3}$.

U|B	*c*_1_	*c*_2_	*c*_3_
*u*_1_	(1,〈0.20,0.80〉)	(2,〈0.60,0.70〉)	(3,〈0.80,0.40〉)
*u*_2_	(2,〈0.70,0.60〉)	(1,〈0.50,0.80〉)	(2,〈0.60,0.70〉)
*u*_3_	(3,〈0.80,0.40〉)	(2,〈0.70,0.60〉)	(2,〈0.60,0.60〉)

$K_{M_{1}}$	(0,〈0.19,0.91〉)	(1,〈0.40,0.90〉)	(2,〈0.60,0.55〉)
$K_{M_{2}}$	(1,〈0.25,0.75〉)	(1,〈0.22,0.78〉)	(3,〈0.80,0.40〉)

**Figure 3:**
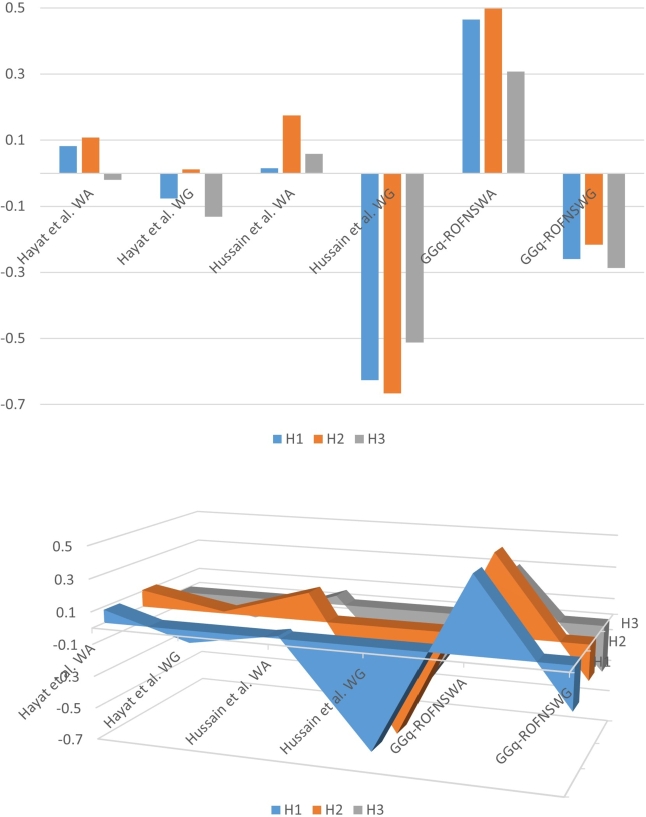
Comparison.

Now applying proposed method for GGq-ROFNSWA, we obtained $WA^{H_{1}}=\langle 0.8288,0.2897\rangle$, $WA^{H_{2}}=\langle 0.8407,0.2947\rangle$, $WA^{H_{3}}=\langle 0.7606,0.4027\rangle$ and score values are $S(WA^{H_{1}})=0.4648$, $S(WA^{H_{2}})=0.4979$, $S(WA^{H_{3}})=0.3084$. The ranking obtained as in following $H_{2}>H_{1}>H_{3}$. The ranking remained the same when the GGq-ROFNSWG operators were applied, that is, $H_{2}>H_{1}>H_{3}$. Using the aforementioned techniques, we arrive at the comparison results displayed in Table 13. Moreover, Fig. 3 also provides a more logical interpretation of the data of Table 13.

**Table 13:** Comparison with existing methods.

Method	Values of Score Function	Ranking
Comparison with Example 4.1		

Hayat et al. [46] (WA)	$S(WA^{H_{1}})=0.0001$, $S(WA^{H_{2}})=-0.0065$, $S(WA^{H_{3}})=-0.0181$, $S(WA^{H_{4}})=-0.0033$	$H_{1}>H_{4}>H_{2}>H_{3}$.
Hayat et al. [46] (WG)	$S(WG^{H_{1}})=-0.1655$, $S(WG^{H_{2}})=-0.1865$, $S(WG^{H_{3}})=-0.2791$, $S(WG^{H_{4}})=-0.2571$	$H_{1}>H_{2}>H_{4}>H_{3}$.
Hussian et al. [22] (WA)	$S(WA^{H_{1}})=0.0430$, $S(WA^{H_{2}})=0.0655$, $S(WA^{H_{3}})=0.04943$, $S(WA^{H_{4}})=0.0189$	$H_{2}>H_{3}>H_{1}>H_{4}$.
Hussian et al. [22] (WG)	$S(WG^{H_{1}})=0.4456$, $S(WG^{H_{2}})=0.4502$, $S(WG^{H_{3}})=0.2532$, $S(WG^{H_{4}})=0.1103$	$H_{2}>H_{1}>H_{3}>H_{4}$.
GGq-ROFNSWA	$S(WA^{H_{1}})=0.1045$, $S(WA^{H_{2}})=0.9085$, $S(WA^{H_{3}})=0.1169$, $S(WA^{H_{4}})=0.0579$	$H_{2}>H_{3}>H_{1}>H_{4}$.
GGq-ROFNSWG	$S(WG^{H_{1}})=-0.5027$, $S(WG^{H_{2}})=-0.4842$, $S(WG^{H_{3}})=-0.5142$, $S(WG^{H_{4}})=-0.9950$	$H_{2}>H_{1}>H_{3}>H_{4}$.

Comparison with Example 4.2		

Hayat et al. [46] (WA)	$S(WA^{H_{1}})=0.0817$, $S(WA^{H_{2}})=0.1081$, $S(WA^{H_{3}})=-0.0198$	$H_{2}>H_{1}>H_{3}$
Hayat et al. [46] (WG)	$S(WG^{H_{1}})=-0.0762$, $S(WG^{H_{2}})=0.0123$, $S(WG^{H_{3}})=-0.1315$	$H_{2}>H_{1}>H_{3}$
Hussian et al. [22] (WA)	$S(WA^{H_{1}})=0.0149$, $S(WA^{H_{2}})=0.1751$, $S(WA^{H_{3}})=0.0591$	$H_{2}>H_{3}>H_{1}$
Hussian et al. [22] (WG)	$S(WG^{H_{1}})=-0.6257$, $S(WG^{H_{2}})=-0.6657$, $S(WG^{H_{3}})=-0.5121$	$H_{3}>H_{1}>H_{2}$
GGq-ROFNSWA	$S(WA^{H_{1}})=0.4648$, $S(WA^{H_{2}})=0.4979$, $S(WA^{H_{3}})=0.3084$	$H_{2}>H_{1}>H_{3}$.
GGq-ROFNSWG	$S(WG^{H_{1}})=-0.2600$, $S(WG^{H_{2}})=-0.2157$, $S(WG^{H_{3}})=-0.2867$	$H_{2}>H_{1}>H_{3}$.

Hayat et al. [46]'s findings are based on GGIVq-ROFSSs, however if we take one membership and one non-membership rather than an interval, this yields GGq-ROFSSs results. Eventually, we compare the aforementioned technique of GGq-ROF4SSs with GGq-ROFSSs by considering memberships and non-membership in Table 10, Table 11, Table 12. Moreover, we applied the Hussian et al. [22] approach for analysis, taking into account q-ROFSSs from the upper regions of Table 10, Table 11, Table 12. Table 13 presents the results of these comparative study's rankings. This study also came to the conclusion that it is crucial to consider non-binary values and other moderator inputs while using GGq-ROF4SSs.

Table 13 and Figure 2, Figure 3 show that despite the fact that the score functions used in the various procedures vary, the final ranking outcomes are substantially the same across all of them. We are aware that closed rankings frequently result in confusion when making the best pick. The higher values of the scores obtained using GGq-ROFNSWA and GGq-ROFNSWG in Figure 2, Figure 3 demonstrate that the distance between options increases and that the optimal solution obtained using this method is consequently more trustworthy. On the other side, relying solely on an object's membership and non-membership can occasionally be insufficient when historical ratings of the things are extremely important. In comparison to GGq-ROFSS or q-ROFSS based results, the usage of GGq-ROFNSSs based outcomes is therefore more significant. On the other hand, when the number of alternatives increases, the given method is helpful to obtain distinct scores to rank alternatives easily.

Regarding the drawbacks, the decision-making method we suggest based on GGq-ROFNSSs can avoid those flaws of the existing decision-making methods, whose primary motive is to take into account the rating levels in a non-binary evaluation environment with a degree of membership and non-membership. The moderators' inputs or evaluations in GGq-ROFNSSs which are important components to summarize data in q-ROFNSSs, but Zhang et al. [35]'s technique is ineffective for any further input. The MCDM using GGq-ROFNSSs offers a methodical approach to deal with uncertainties in cases when alternatives and criteria depend on the final judgments of senior experts and the weights of each component in GGq-ROFNSSs are significant.

## 5 Conclusion

MCDM techniques successfully handle ambiguous and uncertain information in the context of logical decision theory. In many intricate real-world scenarios, alternatives depend not only on criteria but also on underlying sub-alternatives, criteria and rating values. In such situations when there are several granular and interdependent components of data, correct modeling is a difficult step which needed in addition to a well-defined MCDM method. The GGq-ROFNSSs, which are a logical extension of the q-ROFNSSs, give decision-makers a more flexible means of addressing and assessing membership and non-membership values in a non-binary complex MCDM. In contrast to current hybrid models, which use q-ROFNSSs and q-ROFSSs as tools, this research presented a novel hybrid structure called GGq-ROFNSSs, which manage levels in non-binary system with membership and non-membership values.

First, we have introduced N-Pq-ROFS and its associated properties in this study. We introduced GGq-ROFNSSs, distinct inclusion features of GGq-ROFNSSs, weak complement, top weak complement, bottom weak complement, and associated properties of GGq-ROFNSSs based on the concept of N-Pq-ROFS. After GGq-ROFNSSs were introduced, a more adaptable aggregation operator had to be defined in order to use GGq-ROFNSSs in MCDM. We have now given the GGq-ROFNSWA and GGq-ROFNSWG operators as well as their idempotency, monotonicity, and boundedness features. Our MCDM approach for the GGq-ROFNSWA and GGq-ROFNSWG operators has been introduced, and an example of how to choose an appropriate solar panel for a city in various areas is provided. Finally, we have provided a comparison of the suggested method with other ways to demonstrate its advantages.

The GGq-ROFNSSs depiction handles the following: (i) alternatives (a set of solar panel), (ii) sub-alternatives (areas where solar panels are installed), (iii) parameters (a set of environmental effects and power efficiency), and (iv) significant seasonal effects. Our approach of MCDM using GGq-ROFNSSs deals with many granular aspects of complex problems. Therefore, GGq-ROFSSs is a full model that can be used to label every component of MCDM, it is more appropriate to solve MCDM issues by taking into account the q-RONFSSs and extra parameters. The issue addressed by this method has implications for pattern recognition, recommendation systems, and industrial decision-making.

We will analyze interval valued GGq-ROFNSSs, Maclaurin symmetric mean, VIKOR, Dombi operators and aggregation operators in the future and work to remove these theories' shortcomings and roadblocks. Our goal is to use our findings in a variety of domains, including engineering designs, medical sciences and computer sciences. GGq-ROFNSSs theory is very useful and trustworthy for assessing uncomfortable and complex information, however in some real-world scenarios, GGq-ROFNSSs falls short when professionals are confronted with the issue of data sets in triplet, such as data separated into yes, no, and abstinence classes. In these situations, the theory of generalization of picture fuzzy N-soft set and its extensions are necessary. Moreover, in future work we extended our work for rankings similarity in MCDM [47], [50].

## Ethics declaration statement

According to the authors, this is their original research, which hasn't been submitted to or yet being considered by any other journals.

## CRediT authorship contribution statement

**Muhammad Saeed Raja:** Formal analysis, Data curation. **Khizar Hayat:** Writing – original draft, Supervision, Conceptualization. **Adeeba Munshi:** Visualization, Formal analysis. **Tahir Mahmood:** Writing – review & editing. **Rawish Sheraz:** Conceptualization. **Iqra Matloob:** Data curation.

## Declaration of Competing Interest

The authors declare that they have no known competing financial interests or personal relationships that could have appeared to influence the work reported in this paper.

## Data Availability

No data was used for the research described in the article.
